# Formation of Giant Unilamellar Vesicles Assisted by Fluorinated Nanoparticles

**DOI:** 10.1002/advs.202302461

**Published:** 2023-10-09

**Authors:** Jorik Waeterschoot, Willemien Gosselé, Hojjat Alizadeh Zeinabad, Jeroen Lammertyn, Erin Koos, Xavier Casadevall i Solvas

**Affiliations:** ^1^ Mechatronics, Biostatistics and Sensors (MeBioS) at KU Leuven Willem de Croylaan 42 3001 Leuven Belgium; ^2^ Soft Matter Rheology and Technology (SMaRT) at KU Leuven Celestijnenlaan 200J 3000 Leuven Belgium

**Keywords:** artificial cells, giant unilamellar vesicles (GUVs), pickering emulsions

## Abstract

In the quest to produce artificial cells, one key challenge that remains to be solved is the recreation of a complex cellular membrane. Among the existing models, giant unilamellar vesicles (GUVs) are particularly interesting due to their intrinsic compartmentalisation ability and their resemblance in size and shape to eukaryotic cells. Many techniques have been developed to produce GUVs all having inherent advantages and disadvantages. Here, the authors show that fluorinated silica nanoparticles (FNPs) used to form Pickering emulsions in a fluorinated oil can destabilise lipid nanosystems to template the formation of GUVs. This technique enables GUV production across a broad spectrum of buffer conditions, while preventing the leakage of the encapsulated components into the oil phase. Furthermore, a simple centrifugation process is sufficient for the release of the emulsion‐trapped GUVs, bypassing the need to use emulsion‐destabilising chemicals. With fluorescent FNPs and transmission electron microscopy, the authors confirm that FNPs are efficiently removed, producing contaminant‐free GUVs. Further experiments assessing the lateral diffusion of lipids and unilamellarity of the GUVs demonstrate that they are comparable to GUVs produced via electroformation. Finally, the ability of incorporating transmembrane proteins is demonstrated, highlighting the potential of this method for the production of GUVs for artificial cell applications.

## Graphical abstract

A novel method for the production of giant unilamellar vesicles (GUVs) is presented. Here, partially fluorinated silica nanoparticles are utilized for the stabilization of a lipid‐containing aqueous droplet in a fluorinated oil. The encapsulated lipids assemble at the droplet interface forming a GUV, which can be released in an outer aqueous medium by centrifugation.

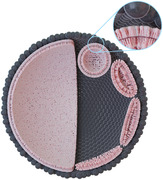

## 1 Introduction

The basic unit of terrestrial life is the cell. These units consist on the compartmentalization of a set of specialized molecules able to catalyze a wide set of reactions (from information processing and replication to energy conversions). In the last few decades, substantial scientific research has focused on recreating these key building blocks of life.^[^ ^1^, ^2^, ^3^, ^4^, ^5^ ^]^ An essential feature for the replication of cellular life processes in an artificial counterpart is the recreation of the outer cell membrane, which typically consists of a bilayer of diverse lipids. This membrane provides both effective compartmentalization and an adequate environment wherein essential biological processes, mainly mediated by transmembrane proteins, can take place. Such artificial lipid bilayers can be mimicked by many models, with giant unilamellar lipid vesicles (GUVs) being the most relevant for generating artificial cells, given the similar sizes and curvatures to those of eukaryotic cells.^[^ ^6^ ^]^

Currently, a wide range of protocols exists to generate GUVs. Earlier methods, such as thin film hydration and electroformation, suffer from low encapsulation efficiencies and do not enable tuning of either the lamellarity or size of the final vesicles. Additionally, electroformation in physiological conditions is difficult since the electric fields cause lipid oxidation and can only incorporate a limited amount of negatively charged lipids (<10%).^[^ ^6^, ^7^, ^8^, ^9^ ^]^ To bypass these issues, techniques like emulsion transfer,^[^ ^10^, ^11^ ^]^ microfluidic jetting,^[^ ^12^ ^]^ and double emulsion droplets^[^ ^13^ ^]^ have been developed, which enable high encapsulation efficiencies. However, these techniques suffer from some limitations: traces of the oil in which the lipids are dissolved are often present in the final GUVs, influencing their properties.^[^ ^14^ ^]^ Furthermore, for microfluidic jetting and the production of double emulsion droplets, the manufacturing and operation of microfluidic chips is necessary, which is difficult and not always reproducible and, consequently, strongly dependent on operator experience.^[^ ^13^, ^15^ ^]^ For a more detailed description of GUV production methods and their limitations, recent reviews can be consulted.^[^ ^6^, ^14^, ^15^ ^]^

**Figure 1:**
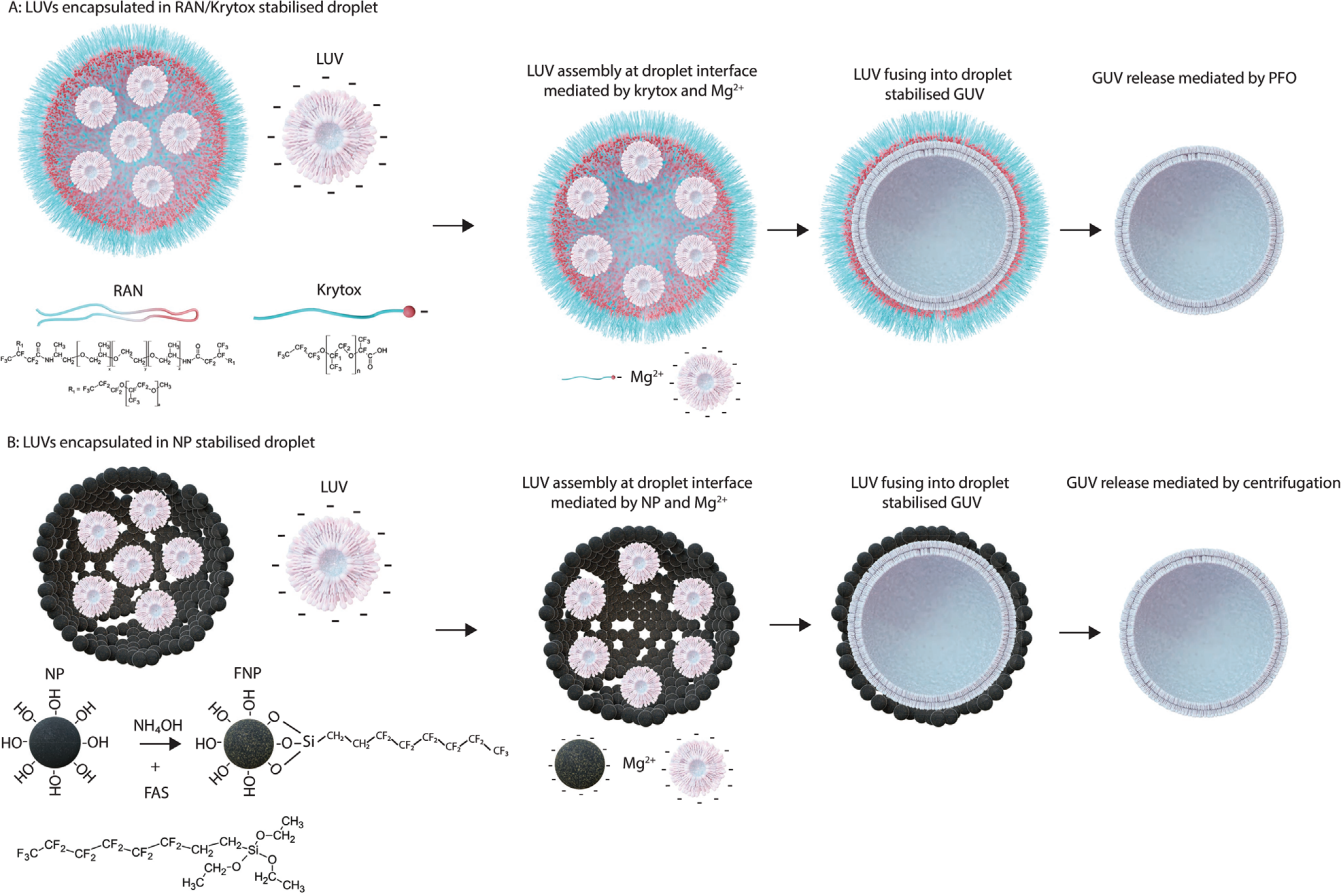
A) GUV formation via the surfactant‐based method developed by Weiss et al.^[^ ^16^ ^]^ A mixture of a PFPE‐PEG‐PFPE block copolymer (RAN) and a PFPE‐carboxylic acid (Krytox) destabilizes LUVs at a droplet interface inside a fluorinated oil phase, resulting in the formation of a droplet‐stabilized GUVs that can be released with the demulsifying agent perfluoro‐1‐octanol (PFO). B) GUV formation method mediated by the FNPs proposed in this paper. Droplets encapsulating LUVs are stabilized by silica particles partially fluorinated with 1H,1H,2H,2H‐perfluorooctyltriethoxysilane (FAS). LUVs are destabilized by particle‐lipid interaction fusing the LUVs into a droplet‐stabilized GUV. Finally, the GUV is released by centrifuging the particles away from the interface of the dsGUV.

Recently, Weiss et al.^[^ ^16^ ^]^ introduced a new GUV production method, based on templating the production of GUVs within surfactant‐stabilized droplets, which act both as a template and a protective environment for the nascent GUV. In this method, an aqueous droplet containing large unilamellar vesicles (LUVs) is stabilized within a fluorinated oil (i.e., HFE 7500 or FC 40) with the help of two different fluorosurfactants: a triblock copolymer consisting of two perfluoropolyether (PFPE) chains linked to a polyethylene glycol (PEG)^[^ ^17^ ^]^ and a PFPE‐carboxylic acid surfactant (i.e., Krytox 157 FSH). In the presence of Mg^2+^ ions, the negative charges of the carboxylic groups on the Krytox surfactant destabilize the LUVs contained within the droplet, resulting in their fusion at the droplet interface and the formation of a droplet‐stabilized GUV with the size of the stabilizing droplet, as illustrated in **Figure** **1**A.^[^ ^16^ ^]^ This method provides high yields and easy encapsulation of biomolecules, which has enabled different applications such as the creation of synthetic organelles,^[^ ^18^ ^]^ the encapsulation of cortical elements (i.e., microtubules and actin),^[^ ^16^, ^19^ ^]^ the production of cytotoxic synthetic T‐cells,^[^ ^20^ ^]^ and the encapsulation of lyotropic liquid crystals.^[^ ^21^ ^]^

Albeit its advantages, the surfactants used in this method strongly interact with the encapsulated molecules and, consequently, mediate undesired transport processes (via micellar or surfactant‐association processes) to the outer oil phase.^[^ ^22^ ^]^ As a result, the encapsulation efficiency of vital cellular biomolecules is dramatically lowered. Furthermore, some biologicallyrelevant molecules will lose their conformation, limiting the complexity of the formed GUV.^[^ ^17^, ^23^, ^24^, ^25^, ^26^, ^27^ ^]^ For example, the encapsulation of actin molecules is not possible without either a second picoinjection step or without being restricted to specific pH‐sensitive lipids.^[^ ^16^, ^19^ ^]^ Additionally, attempts to reconstitute functional transmembrane proteins in GUVs using this method have reported severe interferences with the different components required (surfactants and release chemicals), leading to either the complete removal or inactivation of these sensitive proteins.^[^ ^28^ ^]^ Finally, releasing the GUVs from the droplets in which they are generated is problematic: either specialized microfluidic systems are required (which are complex and low throughput)^[^ ^16^, ^29^ ^]^ or the addition of chemical demulsifiers (e.g., perfluoro‐1‐octanol) is needed, which can interact with hydrophobic residues of proteins destabilizing them.^[^ ^30^ ^]^ All these issues hinder the production of biologically relevant GUVs, precluding their effective utilization as artificial counterparts to actual cells.

Here, we present an alternative method for the production of droplet‐stabilized GUVs by replacing surfactants with nanoparticles (NPs). Our strategy focuses on using partially fluorinated silica nanoparticles (FNPs) to generate 'active' Pickering emulsions, as represented in Figure 1B. Amphiphilic FNPs are obtained via partial functionalisation with 1H,1H,2H,2H‐perfluorooctyltriethoxysilane (FAS), which endows them with the ability to self‐assemble at the liquid–liquid interface between an aqueous solution and a fluorinated oil.^[^ ^31^ ^]^ As these FNPs have a higher adsorption energy at the liquid–liquid interface compared to surfactants (i.e., they are more effectively trapped at the interface), encapsulated biomolecules are more efficiently compartmentalized. Additionally, the hydroxyl groups on the surface of these FNPs, depending on the buffer conditions and the type of lipids, provide a lipid‐destabilizing environment similar to that of traditional silica‐based surfaces used for the formation of solid‐supported lipid bilayers.^[^ ^32^, ^33^, ^34^ ^]^ As such, LUVs fuse on the surface of the FNPs at the droplet's interface forming a GUV. Finally, the GUVs can be released from their encapsulating droplets during a demulsification process consisting of a simple centrifugation step of the Pickering emulsion into an aqueous buffer, which also ensures the effective removal of the FNPs (and, therefore, of the destabilizing reactive groups) without the need to use deleterious chemical demulsifiers.^[^ ^35^ ^]^ All in all, this strategy incorporates the advantages of the droplet‐stabilized method for efficient GUV production without some of its current drawbacks, paving the way to a more effective preparation of artificial cells.

## 2 Results and Discussion

### 2.1 (F)NP Characterization

**Figure 2:**
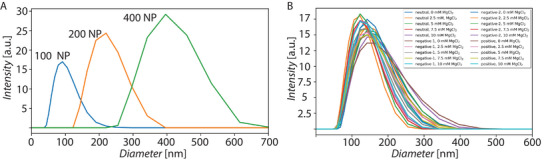
A) NP size distribution for three different particle sizes (mean from three measurements in ethanol). B) LUV size distribution for different lipid compositions under different buffer conditions (mean from three measurements).

To assess the ability of FNPs to assemble at the droplet interface and destabilize LUVs to form droplet‐stabilized GUVs, silica NPs of different sizes, with different fluorination degrees and with different functionalities were created. Three different sizes of silica NPs with diameters of 87 ± 2 nm, 211 ± 1 nm and 393 ± 7 nm were produced via the Stöber method.^[^ ^35^ ^]^ Their size distributions are shown in **Figure** **2**. We will refer to these particles as the 100, 200 and 400 nm particles further in this paper. Figure S1 (Supporting Information) displays the size distribution of the 100 nm FNPs fluorinated with different amounts of FAS (fluorination reagent, see materials and methods section) obtained via DLS. The hydrodynamic diameter remains constant after fluorination except for samples with low amounts of FAS (i.e., the cases of 2.53 · 10^−5^ and 2.53 · 10^−4^ mol FAS/g NPs). Here, the FNPs remain too hydrophilic, leading to their aggregation in HFE oil. Figures S2 and S3 (Supporting Information) show similar results for 200 nm and 400 nm FNPs, respectively, with both sizes retaining similar distributions after fluorination. The fluorination degrees of the 200 and 400 nm FNPs were chosen to result in the formation of stable Pickering emulsions (i.e., no merging of droplets). Lower fluorination degrees resulted in FNP aggregation in HFE oil, while higher fluorination resulted in aggregation of the FNPS in the ethanol medium during their production. For the 100 nm FNPs a more diverse set of fluorination degrees was investigated to assess their behavior at very low and very high fluorination degrees, respectively. In Figure S1 (Supporting Information) the size distribution of rhodamine‐functionalized FNPs is also shown, which remained similar to that of (F)NPs. TEM images in Figure S4 (Supporting Information) confirm the DLS size measurements of the (F)NPs. A negative zeta potential of ‐26 ± 1 mV was measured for the 100 nm NPs (7.58 · 10^−3^ mol FAS/g NPs), which increased after the fluorination reaction to ‐19 ± 1, likely due to a reduction in the number of silanol groups and consequently, of the surface charges. For data on polydispersity indexes (PDI) and diameters see Table S1 (Supporting Information) and PDI (F)NPs.

### 2.2 LUV Characterization

In order to test the ability of our method to produce GUVs from different lipids, LUVs with four different lipid compositions were produced via extrusion: a first mixture mainly consisting of DOPC termed “neutral”, two negatively‐charged mixtures called “negative‐1” and “negative‐2” and one positively charged lipid mixture called “positive” (for the exact composition see the materials and methods section). These LUVs were then encapsulated in droplets stabilized by either surfactants or FNPs to potentially form GUVs. Figure 2B shows the size distributions, which all have a similar peak around 130 nm, confirming that the extrusion process provides homogeneous lipid samples. In Table S2 (Supporting Information) the zeta potential of the four different lipid compositions (neutral, negative‐1, negative‐2 and positive, see methods section for details) in DPBS are shown. As expected, both the negative‐1 and the negative‐2 mixtures have a negative zeta potential (‐21 ± 1 and ‐16 ± 1 mV, respectively) due to the presence of negative PG lipids, the neutral mixture has a neutral zeta potential (0.4 ± 1 mV) and the positive mixture has a positive zeta potential (18 ± 2 mV) as a result of positively charged DOTAP. This potential, the buffer conditions, and the functional groups on the particle surface are the key factors that determine the interaction strength between FNPs and LUVs and will influence GUV formation. For PDI and diameter values of the LUVs, see supplementary material Table S3 (Supporting Information).

### 2.3 (F)NP‐Lipid Interaction

Interactions between silica surfaces and lipid systems like LUVs have been well characterized in the context of the formation of supported lipid bilayers, and include van der Waals, hydration, double‐layer, hydrophobic, thermal undulation, and protrusion forces. Additionally, the eventual fusion of the LUVs interacting with a silica surface is also mediated by vesicle–vesicle interactions.^[^ ^34^, ^36^, ^37^, ^38^, ^39^ ^]^

**Figure 3:**
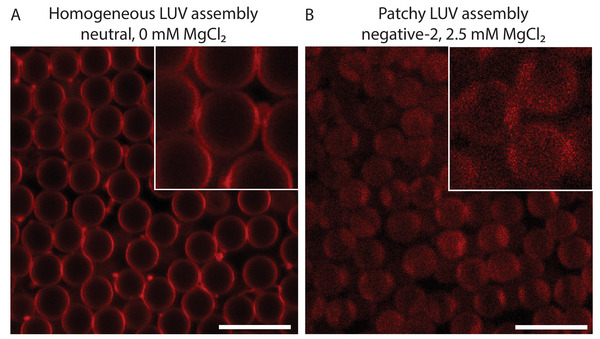
A) Homogeneous lipid assembly of neutral LUVs 0 at mM MgCl_2_ on the surface of 4 µm bare silica particles, evidenced by an even fluorescence intensity across the equatorial plane of the particles. B) Inhomogeneous lipid coverage with negative‐2 LUVs at 2.5 mM MgCl_2_ (scale bar 50 µm, insets 2X magnified).

To investigate the interaction between our LUVs and fluorinated silica surfaces, both bare and fluorinated silica microparticles of 4 µm diameter were incubated with the different LUV solutions used in this study. The fluorination of the microparticles was performed with 5.05 · 10^−4^ mol FAS/g NPs, which is the maximum amount at which particles incubated in an aqueous buffer did not excessively aggregate. Figures S5 and S6 (Supporting Information) show the results for bare and fluorinated silica microparticles, respectively. For bare silica microparticles, LUVs assembled at the interface in all cases except for the negative‐2 LUVs dispersed in a buffer with 0 mM and 2.5 mM MgCl_2_, where a more inhomogeneous coating of the lipids was observed (see **Figure** **3** for examples of both homogeneous and inhomogeneous lipid coverage), potentially due to the high negative zeta potential of the negative‐2 LUVs (−21 ± 1 mV) and the limited ability of Mg^2+^ ions at low concentration to screen these negative charges.^[^ ^34^ ^]^ Similar results have been reported on glass surfaces as the result of the balance between attractive van der Waals forces and repulsive electrostatic forces under specific pH and buffer conditions, which leads to the inhibition of the lipid spreading on such surfaces.^[^ ^38^ ^]^ For the negative‐1 mixture, a higher zeta potential likely results in a lower electrostatic repulsive force, which can be overcome by the zwitterionic attraction force, leading to a more homogeneous spreading of the lipids on the silica surface.

The interaction between the neutral LUVs and silica microparticles can be explained by a van der Waals attraction force. In DOPC, the nitrogen of the headgroup is pointed more outward than the phosphorus atom resulting in a dipole that interacts with the negative charge of the silica particle surface. This interaction is supplemented by the hydrogen bonding with the hydration layer.^[^ ^34^, ^36^, ^37^, ^40^ ^]^ Similarly, the positive lipid mixture is likely attracted to the surface via an extra electrostatic attraction force as a result of the positive charge of the lipids. Given these more robust interactions, these lipids were observed to spread more evenly on the silica microparticles.

For the fluorinated microparticles, the coverage of all lipid systems was observed to be homogeneous. This can be explained by a reduction of the number of hydroxyl groups due to the fluorination, also confirmed by an increase of the zeta potential, decreasing the electrostatic repulsion compared to the van der Waals attraction. These results demonstrate that the diverse lipids from our different LUVs can be effectively assembled on fluorinated silica surfaces.

### 2.4 Droplet Formation

Next, we confirmed the ability of our FNPs to both stabilize droplets and destabilize LUVs encapsulated in these droplets at the droplets' interface, leading to putative formation of GUVs.

#### 2.4.1 High Speed Mixing

**Figure 4:**
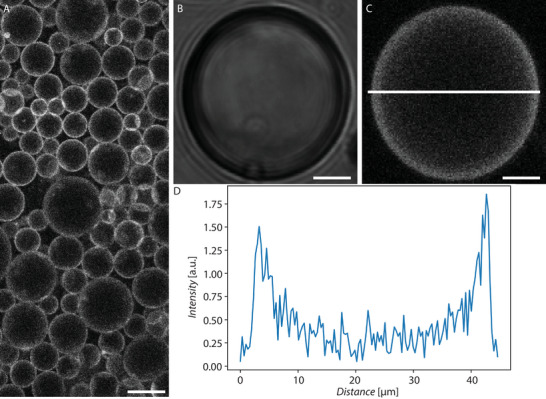
A) Overview image of droplets stabilized by rho‐FNPs (scale bar 50 µm). B) Brightfield image of droplets stabilized by rho‐FNPs imaged in a cell counting chamber. C) Confocal image of droplet in B (scale bars 10 µm). D) Intensity profile of line in B.

In all our experiments, droplets were produced by mixing at high speed (23000 rpm) an aqueous buffer with HFE 7500 oil containing FNPs, delivering a polydisperse droplet population in the size range of interest (5–100 µm), as shown in **Figure** **4**A. Alternatively, droplets could also be produced by vortexing or on a microfluidic chip^[^ ^31^, ^35^ ^]^ (see Figure S7, Supporting Information), but the vortexing method delivered very large droplets (typically >50 µm) and on chip, the number of conditions that could be tested in a time‐efficient manner was limited. Nevertheless, droplets produced with these alternative methods were also efficiently stabilized by the different FNPs.

#### 2.4.2 FNP Localization

Rhodamine‐labeled FNPs (rho‐FNPs) were produced to confirm the distribution of FNPs at a droplet interface. Figure 4A illustrates a typical droplet population produced and imaged with these rho‐FNPs, together with magnified images of one of these droplets (Figure 4B,C). The data show that rho‐FNPs can be used to produce and stabilize droplets. A characteristic fluorescence intensity profile across the equator of one of these droplets (Figure 4D), clearly shows two intensity peaks at the droplet edges, where the droplet interface coincides with the focal plane of the confocal image. The background fluorescence signals inside the droplet can be attributed to scattering as a result of a refractive index mismatch and the presence of free rho‐FNPs being still dispersed in the surrounding fluorinated oil. These results indicate that the FNPs self‐assemble at the droplet interface, since their fluorescence intensity is at its maximum at the edge of the equatorial plane of the droplets, and gradually decays further away from it.

#### 2.4.3 Droplet Formation with LUVs in FNP‐Stabilized Droplets

**Figure 5:**
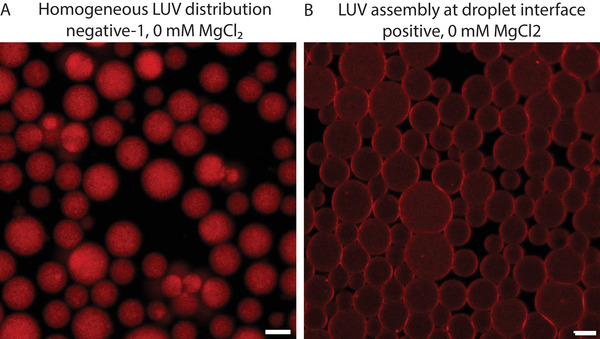
A) Homogeneous lipid distribution of negative‐1 LUVs at 0 mM MgCl_2_ with 100 nm FNP (7.58 · 10^−3^ mol FAS/g NPs). B) Lipids assembled at droplet interface with positive LUVs at 0 mM MgCl_2_ with 100 nm FNP (7.58 · 10^−3^ mol FAS/g NPs) (scale bar 50 µm).

Having assessed the ability of FNPs to self‐assemble at a droplet interface, LUVs from the different lipid mixtures were dispersed in several buffers (see materials and methods section) and subsequently encapsulated in FNP‐stabilized droplets. For 100 nm FNPs fluorinated with 7.58 · 10^−3^ mol FAS/g NPs, in most conditions, LUVs assembled at the droplets' interface, as assessed by the formation of a fluorescent ring of lipids (see figure S8, Supporting Information). Only for the two lowest MgCl_2_ concentrations for both negative mixtures 1 and 2, LUVs remained homogeneously distributed inside the droplets and no rings were formed (see **Figure** **5** for the difference between homogeneous distribution and ring formation). This result was expected since the repulsive forces between both negatively charged LUVs and FNPs cannot be bridged at low Mg^2+^ concentrations. As a result, GUVs could not be produced under these conditions. This behavior is in contrast to the interaction of these lipids with the fluorinated 4 µm silica particles, indicating that the size of the fluorinated particles and/or their equilibration dynamics at the liquid–liquid interface might be important factors affecting LUV destabilization in droplets.

To evaluate the size effect of FNPs, FNPs of different sizes at diverse fluorination degrees were tested (see Figures S9 and S10, Supporting Information). For 100 nm FNPs, LUVs assembled at the droplet interface in all cases (neutral lipids at 0 mM MgCl_2_ and negative‐1 at 5 mM MgCl_2_). This result is similar to the experiment with the 4 µm particles in which both fluorinated and non‐fluorinated microparticles caused lipids from LUVs to assemble at the interface. On the other hand, most droplets stabilized with 200 nm and 400 nm FNPs did not form fluorescent rings (although some smaller droplets did display faint rings), indicating a general lack of capacity of these larger FNPs to attract and destabilize LUVs at the droplet interface. These results could potentially indicate that the relationship between the size of the LUVs and FNPs is of essence: since the 100 nm FNPs are smaller than the 130 nm LUVs used in our experiments, these LUVs might more easily bridge between FNPs to create a more continuous coverage of the droplet interface (i.e. similar to that observed in the 4 µm microparticles or flat silica surfaces). In contrast, when combined with the 200 nm and 400 nm FNPs, the much smaller LUVs encounter an interface which displays a higher apparent roughness. Since roughened silica surfaces have been shown to inhibit the fusion of vesicles and the spreading of their lipids into planar supported lipid bilayers,^[^ ^38^ ^]^ this higher apparent roughness of the interface could explain the different dynamics observed for LUV destabilization at different FNP sizes.

#### 2.4.4 Droplet Formation with LUVs in RAN/Krytox‐Stabilized Droplets

**Figure 6:**
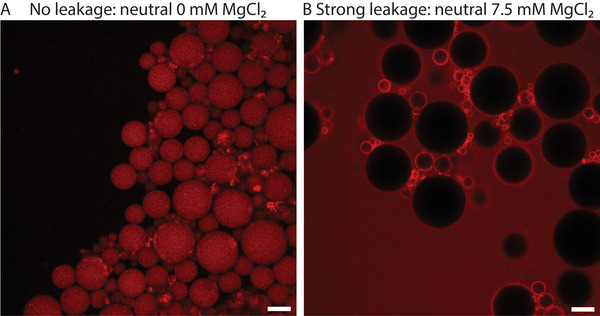
A) Droplets stablized with 3.6% Krytox and 1.4 % RAN with no leakage for neutral LUVs at 0 mM MgCl_2_. B) Droplets stablized with 3.6% Krytox 1.4 % RAN with leakage for neutral LUVs at 7.5 mM MgCl_2_ (scale bar 50 µm).

Next, we compared the performance of our FNP‐based method with the standard droplet‐stabilized strategy for GUV production based on surfactant,^[^ ^16^ ^]^ in terms of leakage of biomolecules. To do so, droplets with the same lipid and buffer compositions were produced in HFE 7500 containing either FNPs or a combination of RAN and Krytox surfactants. Leakage of encapsulated lipids into the oil phase was assessed by measuring the inner fluorescence of at least ten droplets and comparing it to the background fluorescence of the oil phase. For droplets stabilized by FNPs, the outer fluorescence intensity in the oil phase always remained lower than the signal inside the droplets. For surfactant‐stabilized droplets, on the other hand, the opposite happened in multiple conditions, as illustrated in **Figure** **6** for a paradigmatic case (and reported for all cases in Figures S11 and S12, Supporting Information). A high degree of leakage was observed in all cases where rings were formed, which represent the essential conditions required for a sufficient interaction between Krytox and lipids in order to form GUVs.^[^ ^16^, ^41^ ^]^ These results highlight a crucial limitation of the surfactant‐based method for droplet‐stabilized GUV formation, as a large fraction of the biological elements contained in the droplets are lost into the oil phase, hindering the efficient incorporation of biologically relevant species in the GUVs. This is unsurprising, since it has repeatedly been shown that fluorinated surfactants (and specially Krytox) tend to establish strong interactions with a wide range of molecules contained in the dispersed phase and their high mobility leads to the effective removal of these species into the fluorinated continuous phase.^[^ ^25^, ^26^, ^27^ ^]^

#### 2.4.5 GUV Release

After assessing the formation of FNP‐stabilized droplets and their ability to destabilize LUVs at their interface, confirmation of GUV formation (rather than just simple accumulation of lipids at the interface) was sought via attempting the release of these structures into an aqueous buffer. Different methods to destabilize Pickering emulsion were proposed by Pan et al.^[^ ^35^ ^]^ and explored here for the effective release of GUVs from our FNP‐stabilized droplets, including electrocoalescence in bulk, chemical breakup, thermal droplet coalescence and mechanical break‐up by centrifugation at 17 000 g for 15 min (see materials and methods section).

In our case, only the centrifugation method was able to efficiently release GUVs into the outer buffer solution, and only when the speed and time were increased to 100 000 g and 30 min. The droplets produced by Pan et al.^[^ ^35^ ^]^ were larger, around 75 µm, since they were produced by manual shaking. In our case droplets were produced by high speed mixing resulting in a smaller sizes (between 5–50 µm). Since smaller droplets typically are more stable than larger droplets,^[^ ^42^ ^]^ it is likely that in our case larger mechanical forces were necessary to disrupt the droplets' interface and release their contents.

**Figure 7:**
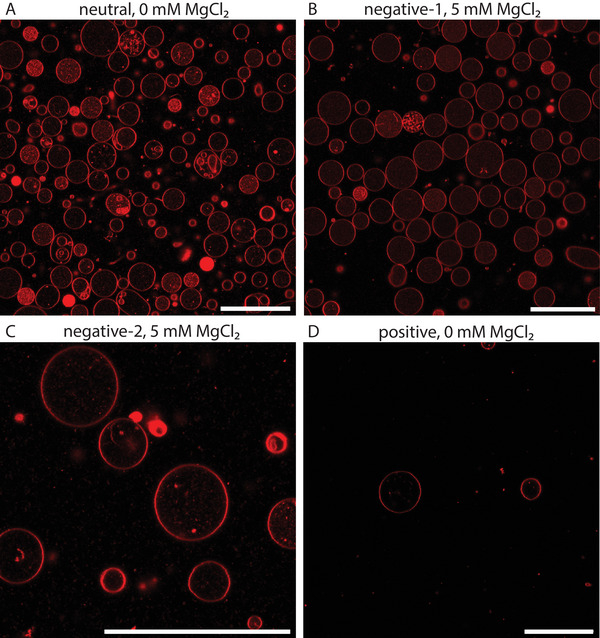
GUVs released under different conditions (scale bar 50 µm). A) neutral, 0 mM MgCl_2_. B) negative‐1, 5 mM MgCl_2_. C) negative‐2, 5 mM MgCl_2_. C) positive, 0 mM MgCl_2_.

Importantly, release of GUVs was only possible in the cases where ring formation was previously observed inside the original droplets, which occured for all our four lipid compositions (see **Figure** **7**). Release from droplets with the negative‐1 and neutral LUVs yielded in general more GUVs (>1000/well) when compared to negative‐2 lipids and the positive lipids (<1000/well).

#### 2.4.6 Release Efficiency

**Figure 8:**
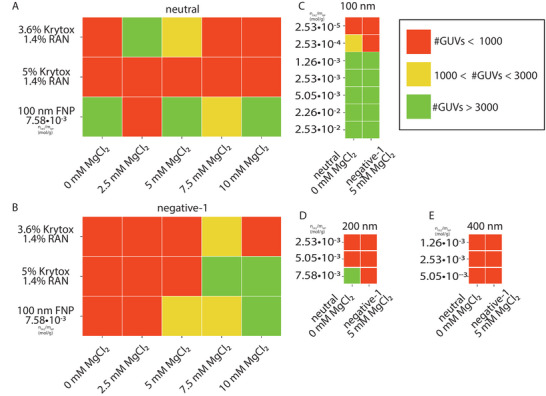
Heatmap of GUVs released for neutral and negative‐1 lipids and counted via an Operetta CLS High Content Analysis System (see materials and methods) for a variety of NP sizes and fluorination degrees. Each result is the average of five repeats. Comparison of GUV release of neutral lipid composition, A), and negative‐1 lipid composition, B), for surfactant‐ and nanoparticle‐based methods for five different magnesium concentrations. C) GUV release for neutral (0 mM MgCl_2_) and negative‐1 (5 mM MgCl_2_) lipid compositions for different fluorination degrees (in mol FAS/g NPs) of 100 nm particles. D) GUV release for neutral (0 mM MgCl_2_) and negative‐1 (5 mM MgCl_2_) lipid compositions for different fluorination degrees of 200 nm particles. E) GUV release for neutral (0 mM MgCl_2_) and negative‐1 (5 mM MgCl_2_) lipid compositions for different fluorination degrees of 400 nm particles.

The GUV release was further analyzed for the neutral and the negative‐1 lipid compositions. The NP‐based method was compared with the surfactant‐based method for different Mg^2+^ concentrations, as illustrated in **Figure** **8**A,B. For the neutral LUVs, the surfactant‐based method was efficient only under one specific condition, whereas our FNP‐based method was very efficient for almost all conditions, except for the 2.5 mM MgCl_2_ case. This is potentially the result of the complex interactions between the different parameters at play (e.g., complex interaction between ionic strength and dissociation constant), which was also previously observed for the surfactant‐based method,^[^ ^41^ ^]^ where only for specific surfactant concentrations a good release was possible. It is also important to highlight that GUVs can be produced with our method in the absence of MgCl_2_, which might be essential for experiments where MgCl_2_ must be avoided. In case of the negative‐1 lipids, release efficiencies were comparable between both methods.^[^ ^41^, ^43^ ^]^

Additionally, the effect of fluorination degree and FNP size was explored, which is illustrated in Figure 8C–E. Based on the DLS data, we confirmed that when FNPs were fluorinated with less or equal than 2.53 · 10^−4^ mol FAS/g NPs, FNPs aggregated in the fluorinated oil. This probably resulted in an inhomogeneous coverage of the droplets with FNPs, hindering effective GUV formation and subsequent release. Higher FNP fluorination did not seem to have any influence on the GUV formation and release efficiency.

In Figure 8D,E it is shown that no GUVs could be released efficiently when FNPs of 200 and 400 nm were used. This was expected, as discussed before, since for these larger FNPs ring formation in droplets was typically not observed, except in droplets of smaller diameters.

#### 2.4.7 Comparison of Droplet and Released GUV Sizes

**Figure 9:**
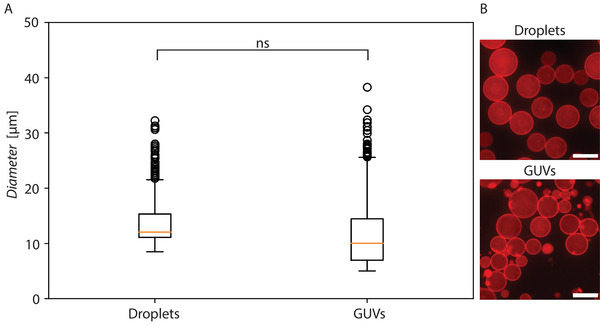
Size comparison between droplets and released GUVs. A) Boxplot of diameters measured from FNP‐stabilized droplets with neutral lipid mixture and corresponding released GUVs (2000 droplets/GUVs were measured in each case and means were compared, p value of 0.05). B) Fluorescence micrograph from samples in A (scale bar 20 µm).

Next, we explored the relationship between the diameter of the droplets produced with our FNP‐based method and the size of the released GUVs. For this, FNP‐stabilized droplets were produced with the neutral lipid mixture (at 0 mM MgCl_2_) and the diameter of 2000 droplets and 2000 released GUVs was measured. As shown in **Figure** **9**, while the mean of the diameter of the droplets and the GUVs did not significantly differ (p value of 0.05), the variance did (p value of 0.05). This is likely the result of the high centrifugation speed needed to release the GUVs, during which a fraction of the GUVs potentially fuses and others potentially split, resulting in a more polydisperse GUV distribution compared to that of the original droplets.

### 2.5 GUV Characterization

#### 2.5.1 Rhodamine FNP on GUV Surface

**Figure 10:**
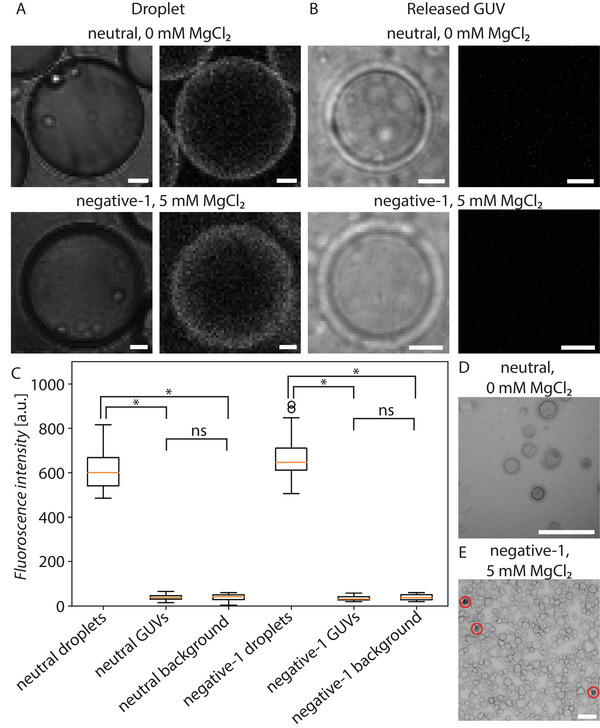
A) Bright field and confocal images of neutral (0 mM MgCl_2_) and negative‐1 (5 mM MgCl_2_) dsGUVs with rho‐FNPs. B) Brightfield and confocal images of released GUVs formed by the droplets in A (scale bar for A and B is 10 µm). C) Boxplot of intensity values measured in droplets, released GUVs and background measured both for neutral and negative‐1 lipids (Ten droplets measured and means compared with p value of 0.05). D) Negatively stained TEM images of neutral sample (0 mM MgCl_2_) in aqueous solution after GUV release. E) Negatively stained TEM images of negative‐1 sample (5 mM MgCl_2_) in aqueous solution after GUV release (scale bar D and E is 500 nm).

To elucidate whether the lipid assemblies released from our FNP‐stabilized droplets were, indeed, GUVs, we first assessed if any FNPs still remained on their surface. We used rhod‐FNPs and assessed the fluorescence signal of rhodamine in both, rho‐FNP‐stabilized droplets and released lipid assemblies, and this signal was compared to the background value (i.e., containing no rho‐FNPs). As shown in **Figure** **10**A–C, the rhodamine fluorescence signal of released lipid assemblies is indistinguishable to that of the background values, indicating that no FNPs are left on the putative GUV surface. To further confirm these results, TEM imaging was performed on negatively stained samples. In most grids, no FNPs could be found, with only a few FNPs visible in one of the samples. It must be noted that GUVs did not survive the TEM imaging and as such, only smaller vesicles are visible in these images. These experiments confirm both that the released lipid assemblies are not a complex structure consisting of FNPs and lipids and that centrifugation is an effective method for releasing these putative GUVs ensuring effective removal of FNPs at the same time.

#### 2.5.2 Lipid Diffusivity

**Figure 11:**
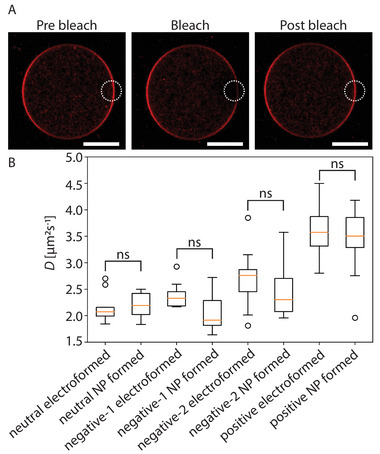
A) FRAP example on neutral GUVs (0 mM MgCl_2_) (scale bar 10 µm). First prebleach image, second an image just after bleaching and third an image of the recovery after 30 s. B) Boxplots of the FRAP values for the different GUV‐like system produced either via electroformation or FNP‐stabilized droplets (ten repeats, means compared with p‐value of 0.05).

To assess the diffusion dynamics of the lipids of the GUVs produced by our method, FRAP experiments were performed on both electro‐ and FNP‐ formed GUVs of identical lipid composition. In **Figure** **11** the data of the two different populations suggest that there is no significant difference between the diffusivity of the lipids formed by these two methods and, therefore, that our method produced GUVs that are free of any major contamination. Additionally, these values are similar to those found in the literature.^[^ ^16^, ^44^, ^45^, ^46^ ^]^

#### 2.5.3 Unilamellarity Assay

**Figure 12:**
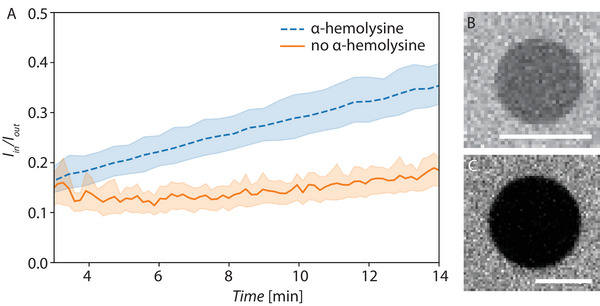
A) Intensity ratio between inner and outer solution analyzed over time with α‐hemolysin (ten GUVs analyzed) and a control without (five GUVs analyzed) upon addition of a fluorescein solution. B) Micrograph after 14 min of incubation in α‐hemolysin and fluoroscein solution. C) Micrograph after 14 min of incubation in a fluoroscein solution. 10 µm scale bar in B and C.

Next, we confirmed the unilamellarity of our putative GUVs using an α‐hemolysin assay (see materials and methods). This pore‐forming protein only assembles in unilamellar lipid bilayers enabling the transport of fluorescent molecules (i.e., fluorescein) that would otherwise not be able to diffuse across the membrane. When added to the outer environment of an empty vesicle, this protein can only incorporate to its outmost bilayer. Therefore, intake of a fluorescent molecule into the vesicle lumen is only possible in unilamellar vesicles. The results of this experiment for our released GUVs are presented in **Figure** **12**, where it can be seen that the fluorescence intensity inside these putative GUVs increases upon addition of fluorescein and α‐hemolysin to the outer buffer solution, confirming their unilamellarity.

#### 2.5.4 Encapsulation

**Figure 13:**
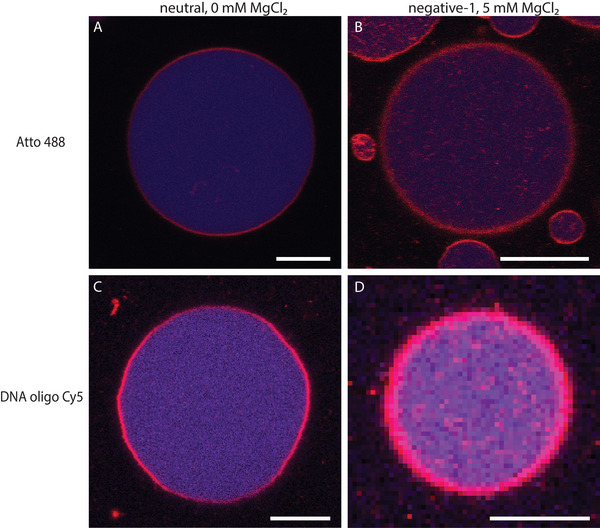
Encapsulation of Atto 488 dye and short cy5‐labeled oligo in neutral (0 mM MgCl_2_) and negative‐1 (5 mM MgCl_2_), 100 nm FNPs with 7.58 · 10^−3^ mol FAS/g NPs (scale bar 5 µm). Blue for the encapsulant and red for the lipids.

**Figure** **13** demonstrates the capability of our method to encapsulate different compounds in GUVs. Both a dye and a dye‐linked oligonucleotide were encapsulated inside GUVs formed with neutral and negative‐1 lipid mixtures, providing proof of principle that encapsulation of biomolecules is possible with our method.

### 2.6 Transmembrane Protein Incorporation

**Figure 14:**
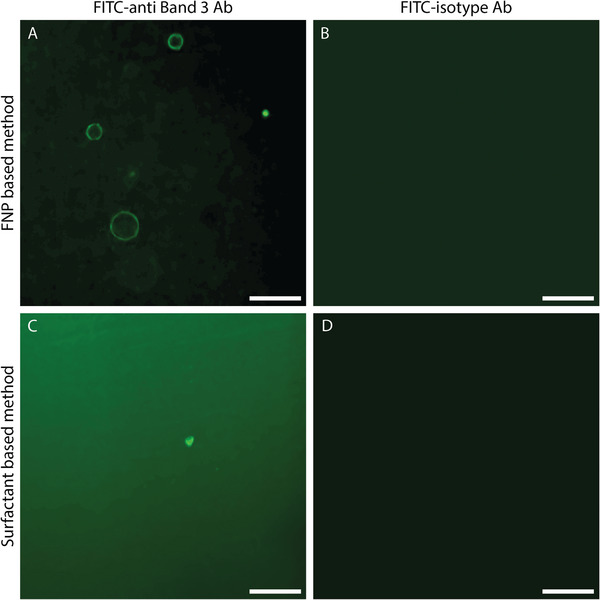
A,B) Micrographs of GUVs produced with proteoliposomes from erythrocytes with the FNP‐assisted method stained with FITC‐anti band 3 Ab (A) and FITC‐isotype Ab (B). C,D) Micrographs of GUVs produced with proteoliposomes from erythrocytes with the surfactant‐based method stained with FITC‐anti band 3 Ab (A) and FITC‐isotype Ab (B). 25 µm scale bar.

Besides encapsulation of biomolecules, to produce functional artificial cells it is of paramount importance to incorporate functional transmembrane proteins, in the lipid environment of the GUVs. To assess the ability of our method to do so, band 3 anion transport protein, an erythrocyte transmembrane protein, was used as a model for complex membrane proteins. Small erythrocyte vesicles containing band 3 were encapsulated and subsequently destabilized inside FNP‐stabilized droplets, and the corresponding GUVs were released and stained with an anti‐band 3 conformational antibody (Ab, with a FITC‐isotype Ab used as a control). In **Figure** **14** it can be observed that the GUVs formed with erythrocyte fragments contained band 3 protein. Since the Ab used was a conformation‐specific Ab for the ectodomain of band 3, at least a fraction of the incorporated band 3 was found both in its correct orientation and native folding configuration. GUVs were not visible after staining with FITC‐isotype Ab indicating that the band 3 Ab specifically interacts with the protein and not with the lipid membrane. Importantly, when the surfactant‐based method was used to attempt the formation of GUVs incorporating band 3 from the same erythrocyte fragments, no GUVs could be formed, highlighting another crucial limitation of this method.

### 2.7 Cost analysis

Finally, an estimation of the cost was carried out, comparing our FNP‐based method with the surfactant‐based method,on the basis of the production of droplets with 0.5 mL of aqueous phase. This comparison is provided in **Table** **1**, which indicates a four times decrease in cost by using FNPs, mainly due to the high cost of the RAN surfactant. For both production methods, HFE 7500 represents a significant cost, which could further be reduced by exploring the use of alternative oils.

**Table 1:** Cost estimation (for 0.5 mL of aqueous solution) for the production of droplets via FNPs and fluorinated surfactants.

NP method	*Cost* [€] / 0.5 mL Aq. phase
TEOS	0.0087
NH_4_OH	0.0050
EtOH	0.3160
FAS	0.1200
HFE 7500	0.6779
Total	1.1275
**Surfactant method**	*Cost* [€] / 0.5 mL Aq. phase
Krytox	0.0290
RAN	3.9033
HFE 7500	0.6779
Total	4.6102

## 3 Conclusion

We have developed a novel method to more effectively harness the formation of biologically relevant GUVs. Our system effectively makes use of fluorinated silica nanoparticles, or FNPs, to produce Pickering emulsions that are capable of destabilizing LUVs at a droplet's interface, resulting in the formation of GUVs. Compared to surfactant‐based strategies for templating GUV formation in droplets,^[^ ^16^ ^]^ our strategy offers several important advantages. First, GUVs could be produced across a broader range of lipids (from four different compositions with negatively, neutrally and positively charged lipids) and buffer conditions. Second, no biomolecules used in the production of these GUVs (lipids, oligos, dyes, and proteins) were observed to leak from the aqueous phase into the continuous oil phase, demonstrating a higher capacity to compartmentalize and effectively utilize such biomolecules. Finally, GUVs were efficiently released into an aqueous buffer by simple centrifugation, which also effectively removed FNPs and eliminated the need to use potentially deleterious chemicals for emulsion break‐up (such as PFO). Release efficiencies between the two methods were comparable, if not slightly better in our case. Furthermore, we found that GUV formation was most effective using FNPs of a smaller diameter (100 nm) than the LUVs (130 nm), with only a limited effect of the fluorination degree. Additionally, our method was estimated to be four times cheaper than the current droplet‐stabilized method. The evidence provided in this study highlights that our method is a more robust alternative for the production of more complex, biologically‐relevant GUVs, with potential applications as artificial cells.

## 4 Experimental Section

### Materials

1,2‐dioleoyl‐sn‐glycero‐3phosphocholine (DOPC), 1‐palmitoyl‐2‐oleoyl‐glycero‐3‐phosphocholine (POPC), 1,2‐dioleoyl‐3‐trimethylammonium‐propane (chloride salt) (DOTAP), 1,2‐Dioleoyl‐sn‐glycero‐3‐phopsho‐(1'‐rac‐glycerol (sodium salt)) (DOPG), Egg phosphatidylcholine (EggPC), Egg phosphatidylglycerol (EggPG), 1,2‐dioleoyl‐sn‐glycero‐3‐phosphoethanolamine‐N‐(lissamine rhodamine B sulfonyl) (ammonium salt) (18:1 Liss Rhod PE), cholesterol, glucose, sucrose, tris(hydroxymethyl)aminomethane (TRIS), TEOS (Tetraethyl orthosilicate), 28% ammonia, (3‐amino propyl)triethoxysilane (APTES), rhodamine B isothiocyanate, perfluoro‐1‐octanol (PFO), bovine serum albumin (BSA), atto 488, fluorescein sodium salt, sodium phosphate monobasic, sodiumphosphate dibasic, EDTA disodium salt and NaCl were obtained from Sigma–Aldrich (Germany). 1H,1H,2H,2H‐perfluorooctyltriethoxysilane (FAS), HFE 7500 from Fluorochem (UK). 4 µm particles were produced by microparticles GmbH (Germany). RAN 008 was obtained from RAN Biotechnologies (US), Krytox 157 FSH from Costenoble (Germany). Absolute Ethanol, Acetone and IPA from Acros organics (Belgium). Uranyl acetate from Electron Microscopy Sciences. Ultrapure water was produced with the Milli‐Q SynErgy UV system from Merck Millipore. Recombinant Staphylococcus alpha Hemolysin protein from Abcam (the Netherlands). DNA oligo (5'‐/5Cy5/CAT CAT CAT CAT CAT CAA A‐3') was obtained from integrated DNA technologies (Belgium),DPBS  from Fisher (UK), magnesium chloride hexahydrate from ThermoFisher (Germany)CD233/Band3 (ext) BRIC 6 FITC test vial from NHS blood and transplants (UK) and FITC Rat IgG1 from BioLegend Europe NV. Erythrocytes were obtained from Red Cross Flanders.

### Methods

**Figure 15:**
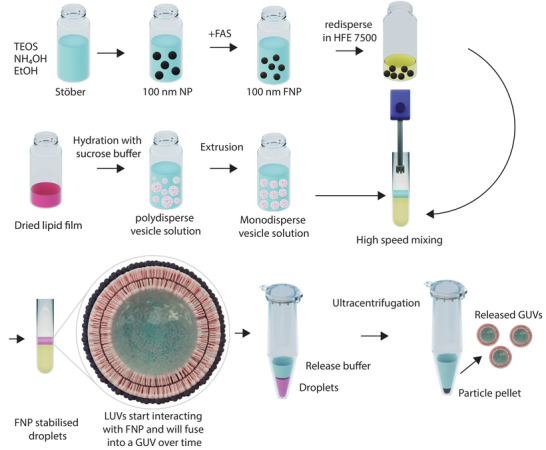
Overview of the proposed method for nanoparticle‐assisted GUV formation.

A general overview of the method is depicted in **Figure** **15**.

### Silica Nanoparticle Production

100 nm silica nanoparticles (NPs) were produced via the Stöber method.^[^ ^47^ ^]^ In short, 3.8 mL of TEOS was dropwise added to 114 mL ethanol and 5.7 mL of 28 (w/w) % ammonium hydroxide. The solution was left stirring overnight. Ammonia was evaporated and particles were stored at 0.01 gmL^‐1^. Alternatively for the 200 nm particles 216 mL ethanol, 9.52 mL ultrapure water, 15.51 mL ammonia hydroxide 28 (w/w) % and 8.79 mL of TEOS were combined. For the 400 nm particles 215.7 mL ethanol, 11.68 mL ultrapure water, 12.18 28 (w/w) % ammonia and 11.06 mL of TEOS. Particles were further modified by reaction with FAS. 200 µl of 28 (w/w) % ammonia solution, 10 mL of 0.01 gmL^‐1^ silica particles and 7.58 · 10^−3^ mol FAS/g NPs (if not mentioned otherwise) was added and the solution was left shaking overnight. Afterwards the particles solutions were transferred to OakRidge centrifuge tubes and spinned down for 30 min at 15 000 g. Particles were resuspended in ethanol by sonicating for 10 min. This cleaning process was repeated three times. Finally, ethanol was removed and replaced with HFE 7500. To remove any leftover ethanol particle solutions were desiccated for at least an hour. To make rhodamine‐labeled NP: first rhodamine‐labeled APTES was produced by mixing 800 mg of APTES and 7.66 mg of rhod B isothiocyanate overnight in 10 mL of ethanol. 100 µl of the solution was mixed with 7.58 · 10^−3^ mol FAS/g NPs as described earlier to produce the rhodB‐labeled particles.

### LUV Production

In the presented work, it was chosen to work with four different lipid compositions, named neutral, negative‐1, negative‐2, positive (%: mol percentage. Neutral (99.5% DOPC, 0.5% liss rhod PE), negative‐1 (34.75% DOPC, 34.75% POPC, 15% DOPG, 15% Cholesterol, 0.5% Liss rhod PE), negative‐2 (80% EggPC, 19.5% EggPG, 0.5% Liss rhod PE), positive (45% DOPC, 45% POPC, 9.5% DOTAP, 0.5% Liss rhod PE). Neutral was chosen as a simple zwitterionic lipid system. Negative‐1 and negative‐2 had been shown to have a relatively high release efficiency as shown in [41, 43]. Positively charged lipids were utilized to show that vesicles could be released from positively charged lipid compositions. LUVs were produced by first transferring the different lipid components to a glass vial with the help of glass Hamilton syringes. Chloroform was evaporated by flushing with nitrogen and dessication for at least two hours. The dried lipid film was hydrated with buffer solution. Different hydration buffers were utilized all containing 30 mM Tris pH 7.5, 300 mM sucrose with different magnesium concentration ranging from 0 to 10 mM MgCl_2_. For the release buffer the sucrose was replaced by 300 mM glucose. Samples were then vortexed for 30 s and sonicated for 30 min. Afterwards samples were extruded nine times through a 50 nm polycarbonate membrane with an Avanti Polar Lipid extruder set. LUV samples were stored in the fridge and utilized within three days of the extrusion process.

### DLS and Zeta Potential

DLS and zeta potential measurements were performed with the zetasizer Nano ZSP (Malvern Panalytical, UK). For zeta potential samples were measured in a disposable capillary cell (DTS1070) at 25°C. DLS samples were measured in a quartz cuvette (ZEN 2112) at 173° angle (25°C). Each sample was measured three times. Following material properties were used: EtOH (refractive index: 1.287, dynamic viscosity: 1.074 cP, dielectric cte: 25.3), HFE 7500 (refractive index: 1.290, dynamic viscosity: 1.240 cP, dielectric cte: 5.8), buffer/PBS (properties of pure water at 25 °C were utilized, refractive index: 1.330, dynamic viscosity: 0.887 cP), lipids (refractive index: 1.450), silica particles (refractive index: 1.540). Zeta potential of LUVs was measured in 1x DBPS.

### TEM

A 300 mesh cupper TEM grid was glow discharged for 15 s. 3.5 µl Sample was pipetted on and incubated for 5 min. For lipid‐containing samples: 30 µl of 1% uranyl acetate was incubated on the grid for 1 min. Samples were blotted and imaged.

### FNP‐Lipid Interaction

4 µm (fluorinated) silica particles were incubated with the different LUV samples. To fluorinate the 4 µm silica particles 1 mL of commercial particle solutions was washed three times in ethanol by centrifuging at 1000 g for 5 min. Particles were redispersed with 5.37 mL ethanol and 153.6 µl 28 (w/w) % ammonium hydroxide. 5.05 · 10^−4^ mol FAS/g NPs was added and samples were left overnight shaking. Fluorinated particles were washed three times with ethanol. For the lipid incubation 25 µl of particles were washed three times with the respective buffer solutions by centrifuging at 1000 g for 5 min. These particles were transferred to 100 µl of 2 mM LUV solution and incubated for at least 3 h on a rotator.

### Droplet Production

FNPstabilized droplets were produced by mixing 500 µl of the LUV‐containing aqueous phase (2 mM lipids) and 250 µl of 0.1 gmL^‐1^ fluorinated nanoparticle solution in 750 µl HFE 7500 with an ultraturax T‐25 at 24 000 rpm for 20s. Droplets were stored overnight in the fridge at 4°C.

RAN/Krytox‐stabilized droplets were produced by mixing 50 µl of the LUV‐containing aqueous phase with 100 µl of the surfactant mixture with a benchtop vortexer for 30 s. After vortexing a white droplet layer was formed on top of the oil phase. Two different mixture of RAN and Krytox were prepared in HFE 7500 oil: 3.6 (w/w) % Krytox with 1.4 (w/w) % RAN, 5 (w/w) % Krytox with 1.4 (w/w) % RAN). These were optimized to give the best possible release.

### GUV Release

GUVs formed in FNP‐stabilized droplets were released by taking 50 µl of droplet solution adding 50 µl of glucose release buffer and centrifugation for 30 min at 4 °C at 100 000 g. Surfactant‐stabilized droplets were released by the addition of 50 µl of glucose release buffer solution and 50 µl of a 20 (v/v%) PFO solution. These droplets were stored in the fridge for three hours before imaging.

*Other Techniques Attempted* Electrocoalescence in bulk was performed in an Eppendorf tube with a ring shaped outer electrode made of aluminium foil on the outside and a copper electrode inserted inside the Eppendorf tube (both coated with PDMS to prevent electrolysis) at AC voltages up to 200 V and frequencies up to 1 kHz as proposed by Pan et al.^[^ ^35^ ^]^ Chemical breakup with perfluoro‐1‐octanol (PFO): 50 µl from 20% to a 100% PFO solution was added to 50 µl of droplets. Thermal droplet coalescence by freeze‐thawing was performed by freezing the droplets at ‐18 °C for 1 h followed by a subsequent thawing step.

### Release Efficiency

For the neutral and the negative‐1 lipid composition, the release efficiency was determined for all the different NP sizes and fluorination degrees. This experiment was performed by using the Operetta CLS High Content Analysis System as described in the imaging section. Every condition was repeated five times. Images were segmented and the number of GUVs was counted with the Nikon analysis software. A distinction between three categories was made to categorize the release efficiency. Less than 1000 GUVs released, some GUVs in every image but overall limited. Between 1000 and 3000 GUVs, GUVs clearly present in the sample in large quantities. More than 3000 GUVs, a very good release, almost the whole bottom of the well was covered with GUVs.

### Droplet and GUV Size Analysis

Neutral lipid LUVs were encapsulated in FNP‐stabilized droplets and released by centrifugation as described earlier. The diameters of 2000 droplets and 2000 GUVs were measured with the help of the Nikon analysis software. The results were plotted in a boxplot and mean and variance were compared.

### Rhodamine FNP on GUV Surface

Fluorescence intensity of GUVs, before and after release was analyzed in at least 20 droplets and 20 GUVs for both the neutral and negative‐1 lipid compositions, droplets were formed with rhodamine‐linked fluorescent NPs. These values were compared to the background value. Imaging parameters were kept constant during both droplet and GUV imaging (for these experiments LUVs were not stained with rhodamine).

### Electroformation

10 µl of a 1.5 mgmL^‐1^ lipid chloroform solution was deposited on the conductive side of an ITO coated slide, which was afterwards dessicated overnight. A rubber ring was attached to the slide with some wax around the lipid solution. Lipids were hydrated with 250 µl of a 300 mM sucrose solution and a second ITO coated slide was deposited on top. The slide sandwich was inserted into the vesicle prep pro device (Nanion, Germany). 3 V was applied at a frequency of 5 Hz for 160 min at 36 °C. Temperature was ramped from room T to final temperature over 30 min. Amplitude was first slowly increased from 0.1 to 0.5 V for 30 min and then to 3 V in 15 min. At end of the electroformation, voltage was ramped down to 0 V in 5 min. GUVs were collected and stored in the fridge for maximum one day. 500 µl of a 300 mM glucose solution was added before GUVs were transferred to the imaging chambers.

### Imaging

Confocal Imaging was performed with the Nikon TiE A1R. Droplets were imaged by transferring 8 µl of droplet solution into a cell counting chamber (Kisker Biotech GmbH, Germany). Single GUV samples were imaged in 16 well uncoated polymeric Ibidi slides (Ibidi GmbH, Germany). Each well contained 100 µl of release buffer and 50 µl of GUV solutions. For high throughput experiments a Perkin Elmer cell carrier ultra 96 well plate was used. This plate was coated with BSA by incubating 10 mgmL^‐1^ of BSA in DPBS solution overnight. Afterwards all wells were washed with DPBS and ultrapure water and filled with 100 µl of glucose release buffer, images were collected with the Operetta CLS High Content Analysis System 20x water immersion lens. Per well 69 images were taken in confocal mode, effectively imaging the whole well. All GUV solutions were handled with extra wide orfice pipette tips to reduce applied shear forces. 4 µm silica particles were imaged by transferring 10 µl of the particle solution in 100 µl of clean sucrose buffer solution in an ibidi slide.

### FRAP

GUVs were left for half an hour to sink to the bottom of the Ibidi slide. The center plane of the GUVs was brought in focus of the 60x lens of the Nikon TiE A1R. Next a 5 µm bleaching spot was defined at the edge of the GUV. At least five pre‐bleaching images were collected, afterwards the spot was bleached for 1.2 cycles at full laser power and recovery images were collected for 30 s. Image sequences were normalized using the built‐in function of the Nikon software with background and reference point correction. Recovery time calculation were performed by the Nikon software. Next diffusion constants were calculated with Equation (1),^[^ ^46^ ^]^ $r_{n}^{2}$ as radius of the bleach spot and $\tau_{\frac{1}{2}}$ as recovery time. Average and standard deviation of at least nine samples were calculated and compared for both electroformed and FNP‐formed GUVs.

(1) $$D=\frac{r_{n}^{2}}{\tau_{\frac{1}{2}}}$$

### Unilamellarity Assay

10 µl of neutral GUV solution was transferred to 100 µl of glucose buffer solution in an ibidi slide. The sample was left for half an hour to let the GUVs sediment. 50 µl of a 70 µM fluoroscein in glucose solution with or without α‐hemolysin was added. In case of α‐hemolysin addition the final concentration of the α‐hemolysin was 225 nM. Samples ware imaged for at least 14 min, fluorescence intensity of 10 GUVs was analyzed for the α‐hemolysin, 5 GUVs were analyzed in the control case. The ratio between the inner and outer solution over time was measured.

### Encapsulation

1 µl of cy5 oligo stocksolution was added to 100 µl of sucrose buffer solution with DOPC or Mix LUVs (containing 0 mM MgCl_2_ and 5 mM MgCl_2_, respectively). Atto 488 was encapsulated at a concentration of 5 µM in DOPC and Mix GUVs.

### Transmembrane Protein Incorporation

Proteoliposomes (PLs) enriched with Band 3 anion transport protein were isolated from human erythrocytes (according to [48]). Briefly, human erythrocyte extract was diluted with DPBS in volumetric ratios 2:1 (erythrocyte:DPBS). Samples were centrifuged for 5 min at 1000 g (4 °C). This process was repeated three times. The pellets were redispersed in low ionic strength solution (5 mM sodium phosphate, 0.1 mM EDTA, pH 8) and incubated at RT for 15 min. Next, samples were centrifuged at 27 000 g for 15 min at 4 °C, and were washed two more times. The pellets were then redispersed in very low ionic strength buffer (0.3 mM sodium phosphate, 0.1 mM EDTA, pH 8) and incubated at 43 °C for 30 min followed by centrifugation at 17 000 g for 10 min at 4 °C. The resulting ghosts were washed with saline buffer (50 mM sodium phosphate, 300 mM NaCl, pH 8) and sonicated for 10 min at RT before being passed 11 times through a 100 nm polycarbonate membrane with a mini extruder (Avanti). As a result, monodisperse erythrocyte‐derived proteoliposomes were obtained.

These proteolipsomes were encapsulated in FNP‐ (100 nm,7.58 · 10^−3^ mol FAS/g NPs) and surfactant (3.6% krytox and 1.4% RAN)‐stabilized droplets as described before in a 10 mM MgCl_2_ solution. These droplets were then released and washed with Ab solution, by first centrifuging at 12 000 g for 10 min with release buffer and then incubating with either FITC‐conjugated anti‐Band 3 Ab or a FITC‐tagged isotype Ab (as a negative control) for 30 min on ice in the dark with gentle mixing each 10 min. Finally, GUVs were washed with the release buffer followed by imaging.

### Buffer Preparations

All buffer solutions were prepared in ultrapure water with 30 mM Tris and adjusted to pH 7.5, with 300 mM sucrose or glucose for the preparation of LUVs or the release buffer, respectively This provided an inner sucrose phase and outer glucose phase resulting in the sedimentation of the GUVs, fascilitating their imaging. The concentration of MgCl_2_ was defined in every subsection. For the fluorescein buffer the glucose buffer was complemented with 70 µM of fluorsecein sodium salt.

### Statistical Analysis

The number of repeats for each experiment was stated in the materials and methods of each section. When two means were compared, normal distribution and equal variance conditions were investigated (p‐value 0.05) with the Shapiro‐Wilk and Levene test, respectively. In case of normally distributed data with equal variance the two‐sided student t‐test was utilized (p‐value 0.05). In cases of non‐normal distributed data or unequal variance the Mann‐Whitney test (p‐value 0.05) was utilized.

## Conflict of Interest

The authors declare no conflict of interest.

## Supporting information

Supporting Information

## Data Availability

The data that support the findings of this study are available from the corresponding author upon reasonable request.
