# Supplementary material for: Formation of Giant Unilamellar Vesicles Assisted by Fluorinated Nanoparticles
Source: Adv Sci (Weinh). 2023 Oct 9;10(34):2302461. doi: 10.1002/advs.202302461 (PMC10700689; doi:10.1002/advs.202302461)
Supplement: Supplementary file 1 — Supporting Information [file ADVS-10-2302461-s001.pdf]

ADVANCED SCIENCE
Open Access

Supporting Information

for *Adv. Sci.*, DOI 10.1002/advs.202302461

Formation of Giant Unilamellar Vesicles Assisted by Fluorinated Nanoparticles

Jorik Waeterschoot, Willemien Gosselé, Hojjat Alizadeh Zeinabad, Jeroen Lammertyn, Erin Koos and Xavier Casadevall i Solvas**

Supplementary materials: Formation of Giant Unilamellar Vesicles Assisted by Fluorinated Nanoparticles

Jorik Waeterschoot Willemien Gosselé Hojjat Alizadeh Zeinabad Jeroen Lammertyn Erin Koos Xavier Casadevall i Solvas**

Jorik Waeterschoot
Email Address: jorik.waeterschoot@kuleuven.be Address: Mechatronics, Biostatistics and Sensors (MeBioS) at KU Leuven, Willem de Croylaan 42, 3001 Leuven Belgium
Willemien Gosselé
Email Address: willemien.gossele@kuleuven.be Address: Mechatronics, Biostatistics and Sensors (MeBioS) at KU Leuven, Willem de Croylaan 42, 3001 Leuven Belgium
Dr. Hojjat Alizadeh Zeinabad
Email Address: hojjat.alizadehzeinabad@kuleuven.be Address: Mechatronics, Biostatistics and Sensors (MeBioS) at KU Leuven, Willem de Croylaan 42, 3001 Leuven Belgium
Prof. Jeroen Lammertyn
Address: Mechatronics, Biostatistics and Sensors (MeBioS) at KU Leuven, Willem de Croylaan 42, 3001 Leuven Belgium
Prof. Erin Koos
Address: Soft Matter, Rheology and Technology (SMaRT) at KU Leuven, Celestijnenlaan 200J, 3000 Leuven Belgium
Prof. Xavier Casadevall i Solvas
Email Address: xevi.casadevall@kuleuven.be Address: Mechatronics, Biostatistics and Sensors (MeBioS) at KU Leuven, Willem de Croylaan 42, 3001 Leuven Belgium

Table S1: (F)NP diameter and PDI (mean from three measurements)

	Diameter [nm]	*PDI*
100 nm SiO_2 EtOH	87 ± 2	0.1 ± 0.02
100 nm SiO_2 1 µl	4521 ± 1424	0.5 ± 0.2
100 nm SiO_2 10 µl	605 ± 16	0.3 ± 0.04
100 nm SiO_2 50 µl	94.8 ± 0.6	0.04 ± 0.02
100 nm SiO_2 100 µl	92.5 ± 0.6	0.02 ± 0.01
100 nm SiO_2 200 µl	92.6 ± 0.3	0.02 ± 0.02
100 nm SiO_2 300 µl	89.3 ± 0.4	0.16 ± 0.01
100 nm SiO_2 500 µl	93.4 ± 0.5	0.02 ± 0.01
100 nm SiO_2 1000 µl	93 ± 0.6	0.02 ± 0.01
100 nm SiO_2 Rhodamine	122.4 ± 0.8	0.065 ± 0.002
200 nm SiO_2 EtOH	211 ± 1	0.03 ± 0.02
200 nm SiO_2 100 µl	219 ± 3	0.01 ± 0.01
200 nm SiO_2 200 µl	207 ± 2	0.03 ± 0.01
200 nm SiO_2 300 µl	224 ± 4	0.07 ± 0.03
400 nm SiO_2 EtOH	393 ± 7	0.04 ± 0.02
400 nm SiO_2 50 µl	430 ± 10	0.20 ± 0.01
400 nm SiO_2 100 µl	430 ± 6	0.12 ± 0.06
400 nm SiO_2 200 µl	431 ± 4	0.08 ± 0.04

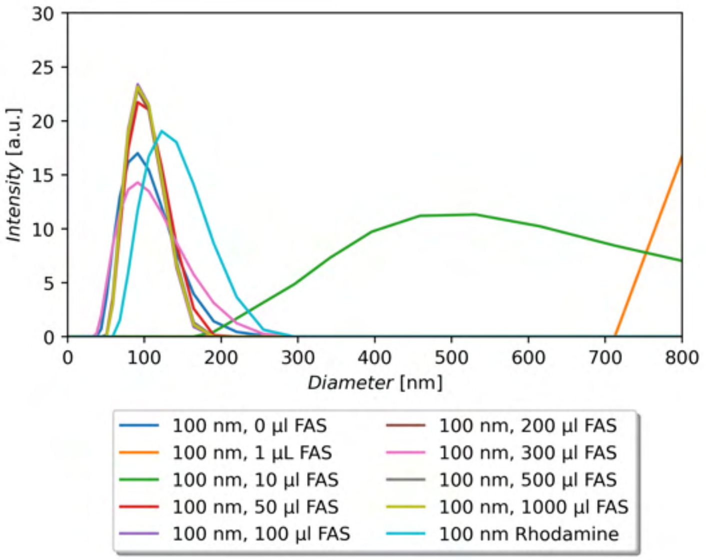

Figure S1: 100 nm FNPs size distributions (mean from three measurements in HFE oil). different amounts of FAS were used for the fluorination. In case of the rhodamine samples were fluorinated with $7.58 \cdot 10^{-3}$ mol FAS/g NPs FAS and 100 µl rhodamine APTES.

Table S2: Zetapotential lipid samples (Mean from three measurements)

	Zp [mV]
neutral	0.4 ± 1
negative-1	-16 ± 1
negative-2	-21 ± 1
positive	18 ± 2

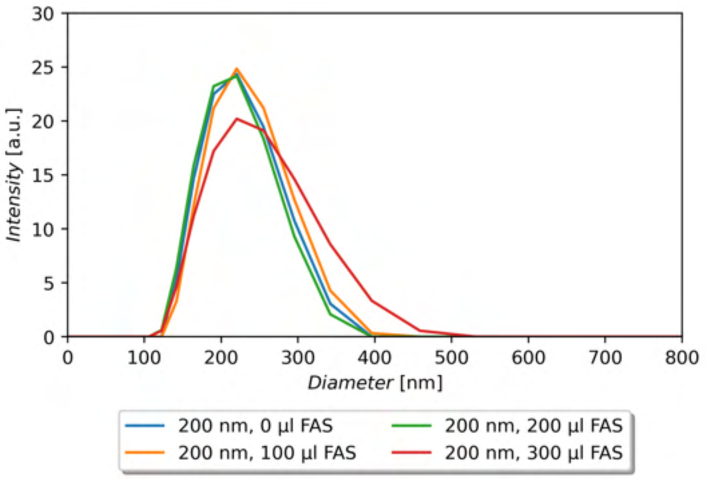

Figure S2: 200 nm FNPs size distributions (mean from three measurements in HFE oil).

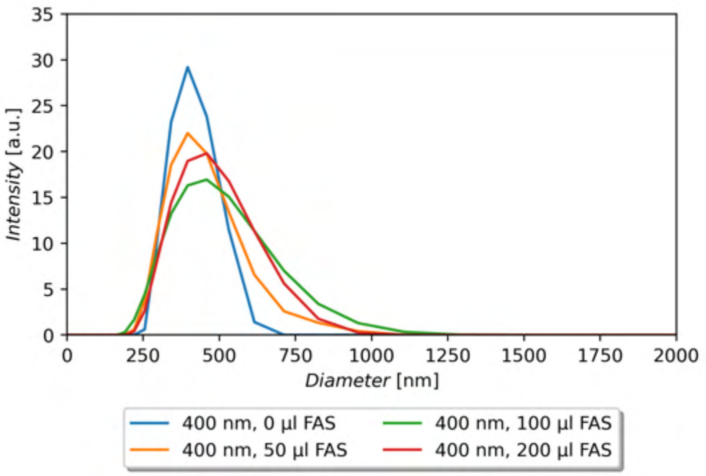

Figure S3: 400 nm FNPs size distributions (mean from three measurements in HFE oil).

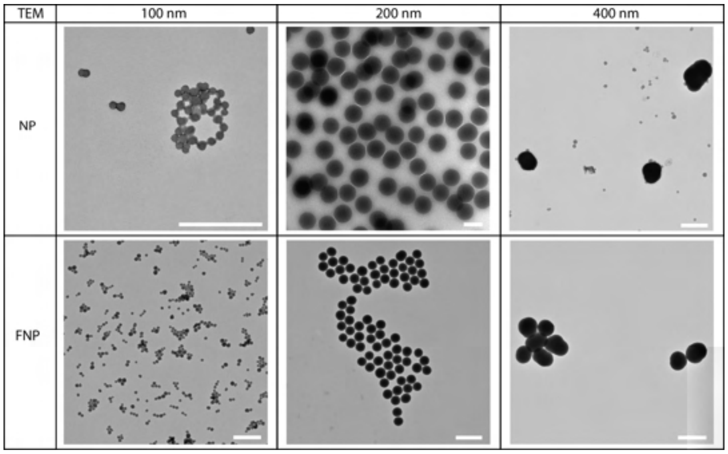

Figure S4: TEM images of different (F)NP (500 nm scale bar).

Table S3: LUV diameter and PDI (Mean from three measurements)

	Diameter [nm]	*PDI*
neutral 0 mM $MgCl_2$	140 ± 1	0.11 ± 0.01
neutral 2.5 mM $MgCl_2$	139 ± 1	0.094 ± 0.004
neutral 5 mM $MgCl_2$	142 ± 1	0.109 ± 0.009
neutral 7.5 mM $MgCl_2$	145 ± 0.9	0.12 ± 0.02
neutral 10 mM $MgCl_2$	145 ± 0.9	0.12 ± 0.02
negative-1 0 mM $MgCl_2$	132.4 ± 0.8	0.12 ± 0.01
negative-1 2.5 mM $MgCl_2$	121.2 ± 0.6	0.09 ± 0.02
negative-1 5 mM $MgCl_2$	129.1 ± 0.5	0.1 ± 0.01
negative-1 7.5 mM $MgCl_2$	131.6 ± 0.3	0.1 ± 0.02
negative-1 10 mM $MgCl_2$	133.1 ± 0.9	0.097 ± 0.003
negative-2 0 mM $MgCl_2$	138.1 ± 0.4	0.09 ± 0.01
negative-2 2.5 mM $MgCl_2$	113.1 ± 0.1	0.09 ± 0.01
negative-2 5 mM $MgCl_2$	119.6 ± 0.8	0.079 ± 0.005
negative-2 7.5 mM $MgCl_2$	119.3 ± 0.5	0.092 ± 0.008
negative-2 10 mM $MgCl_2$	123.8 ± 0.3	0.08 ± 0.02
positive 0 mM $MgCl_2$	144 ± 2	0.13 ± 0.01
positive 2.5 mM $MgCl_2$	129.9 ± 0.6	0.12 ± 0.02
positive 5 mM $MgCl_2$	124.5 ± 0.4	0.1 ± 0.01
positive 7.5 mM $MgCl_2$	131.4 ± 0.4	0.127 ± 0.006
positive 10 mM $MgCl_2$	123.6 ± 0.5	0.106 ± 0.009

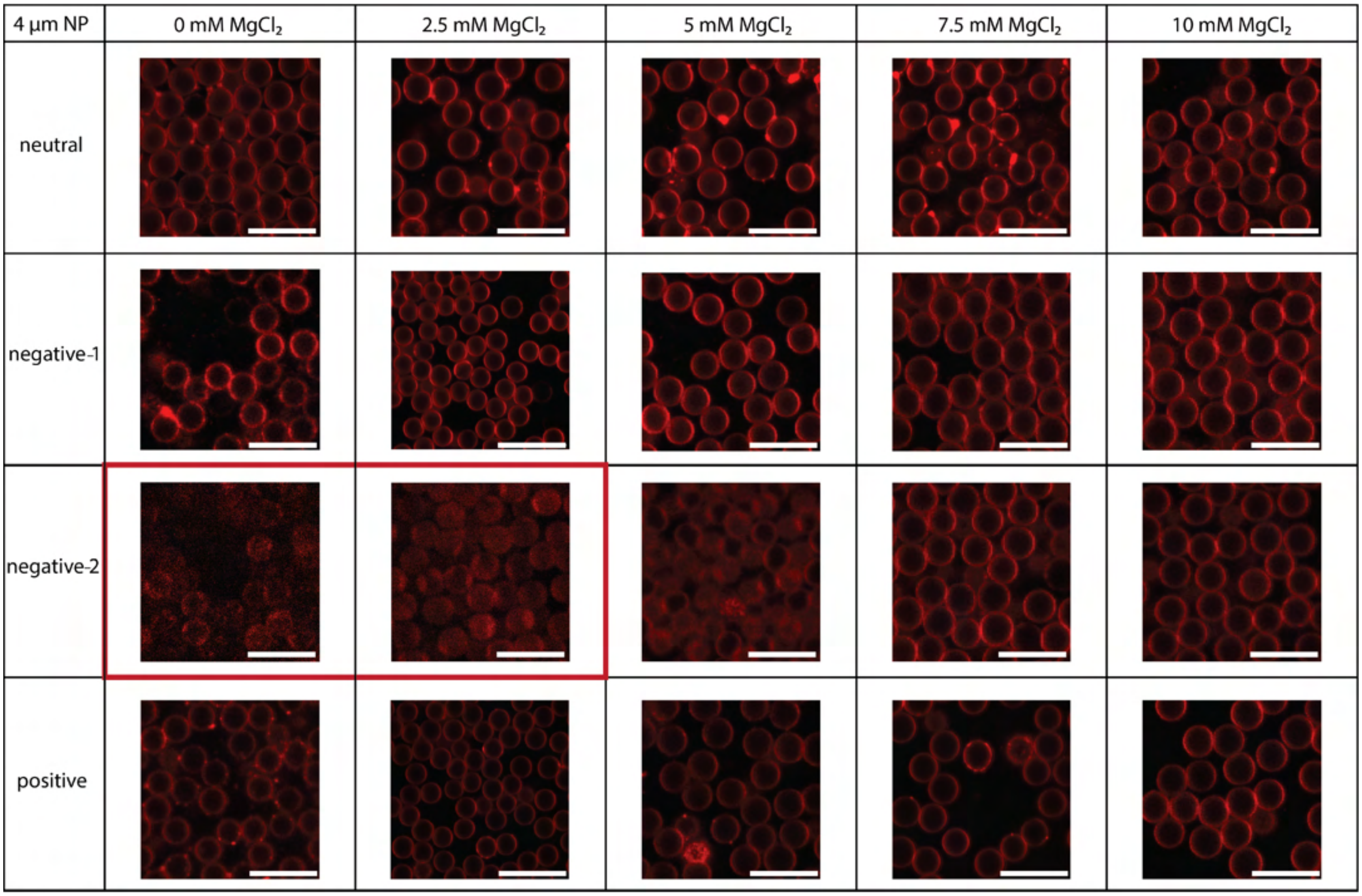

Figure S5: 4 µm microparticles incubated with different LUV solutions imaged in chamber slides. The red square indicates non homogenous particle lipid interaction (10 µm scale bar).

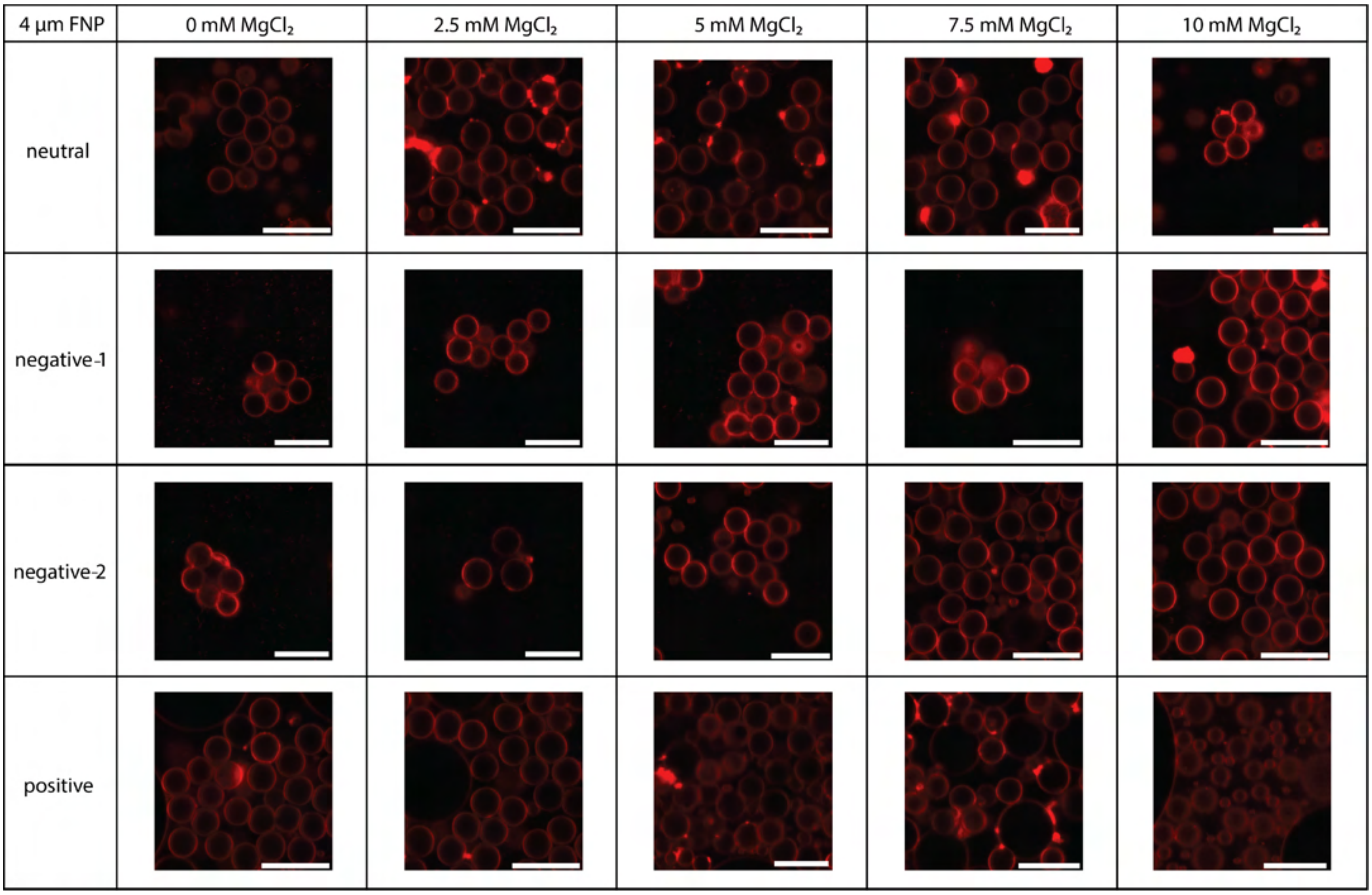

Figure S6: 4 µm fluorinated microparticles incubated with different LUV solutions imaged in chamber slides (10 µm scale bar).

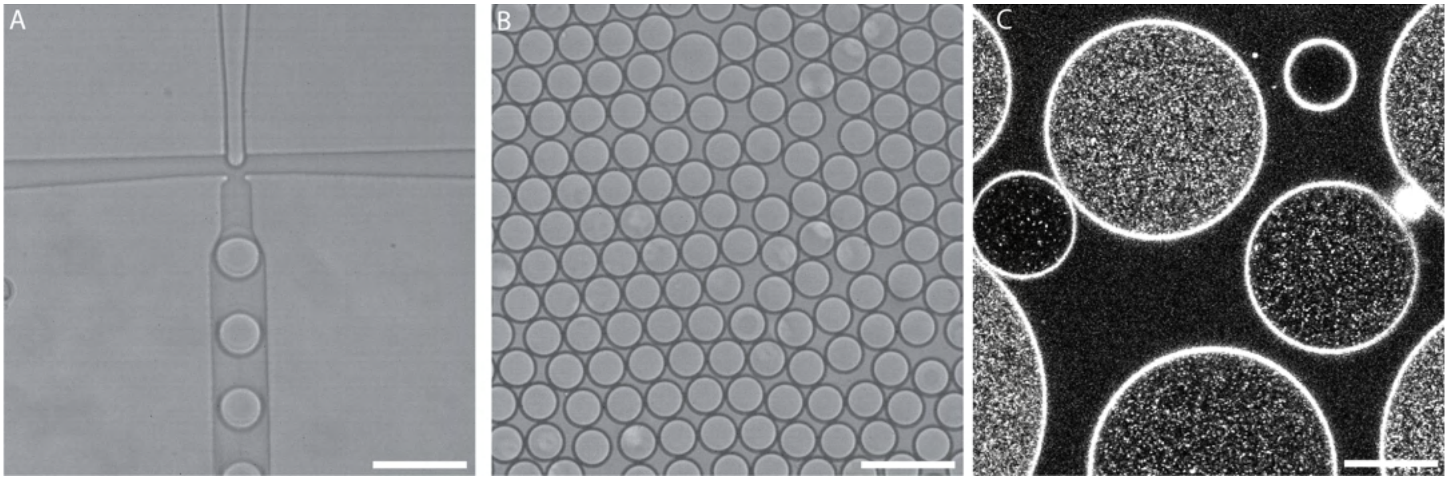

Figure S7: A: Micrograph of flow focussing droplet microfluidic chip producing aqueous droplets stabilized by FNPs. B: Micrograph of droplets produced by microfluidic chip in A collected in cell counting chamber. C: Droplets produced by manual shaking visualised by encapsulating neutral LUVs (glucose buffer 0 mM $MgCl_2$), droplets tend to be larger than 50 µm (100 µm scale bar).

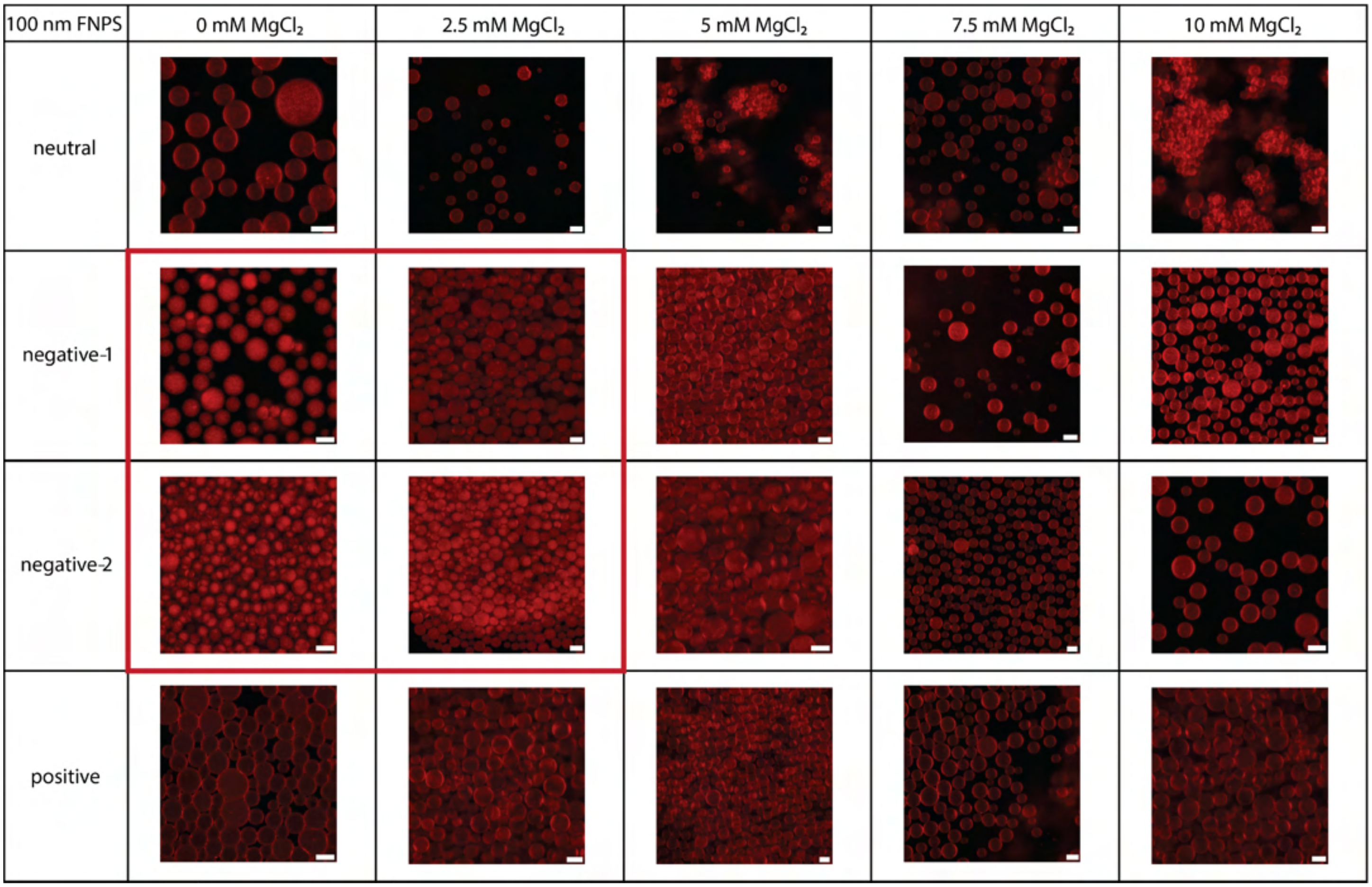

Figure S8: Droplets with different LUV compositions stabilized by 100 nm FNPs fluorinated with $7.58 \cdot 10^{-3}$ mol FAS/g NPs (50 µm scale bar). The red square indicates the conditions in which lipids did not assemble at the droplet interface but remained homogeneously distributed

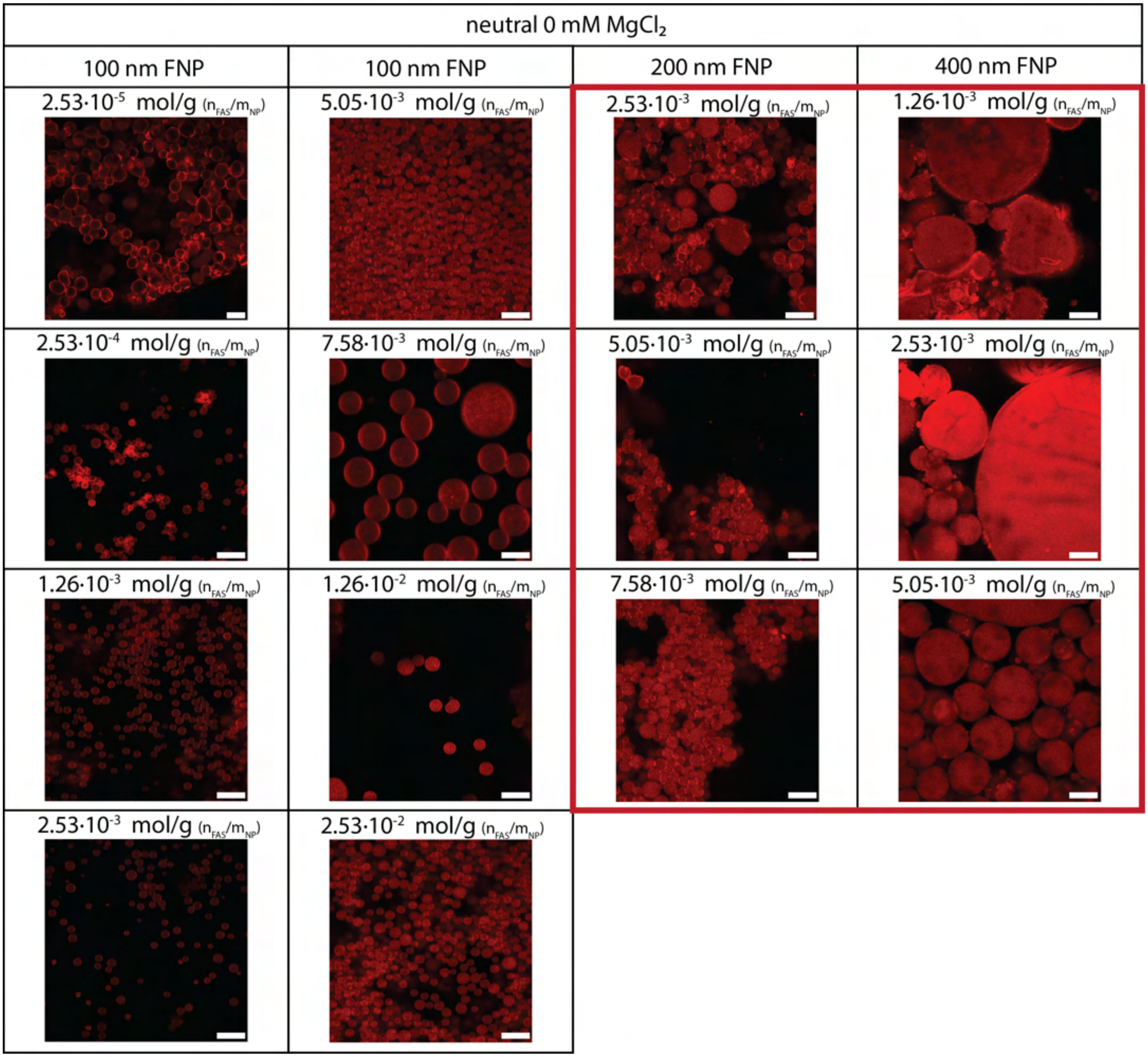

Figure S9: Droplets formed with neutral LUVs with different FNP sizes and fluorination degrees. The red square indicate droplets in which lipids did not assemble at the droplet interface but remained homogeneously distributed (scale bar 50 µm).

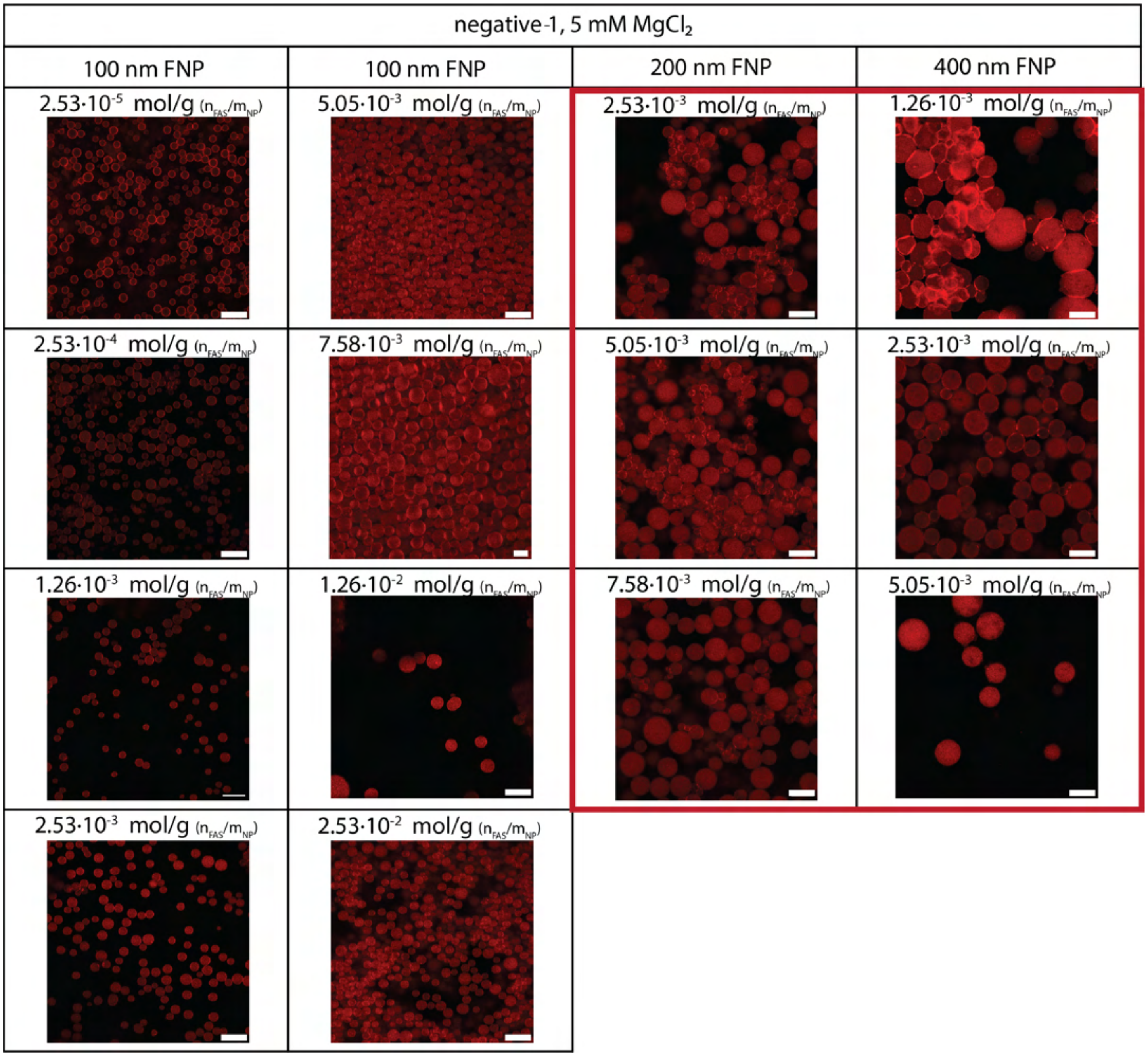

Figure S10: Droplets formed with negative-1 LUVs with different particle sizes and fluorination degrees. The red square indicates droplets in which lipids did not assemble at the droplet interface but remained homogeneously distributed (scale bar 50 µm).

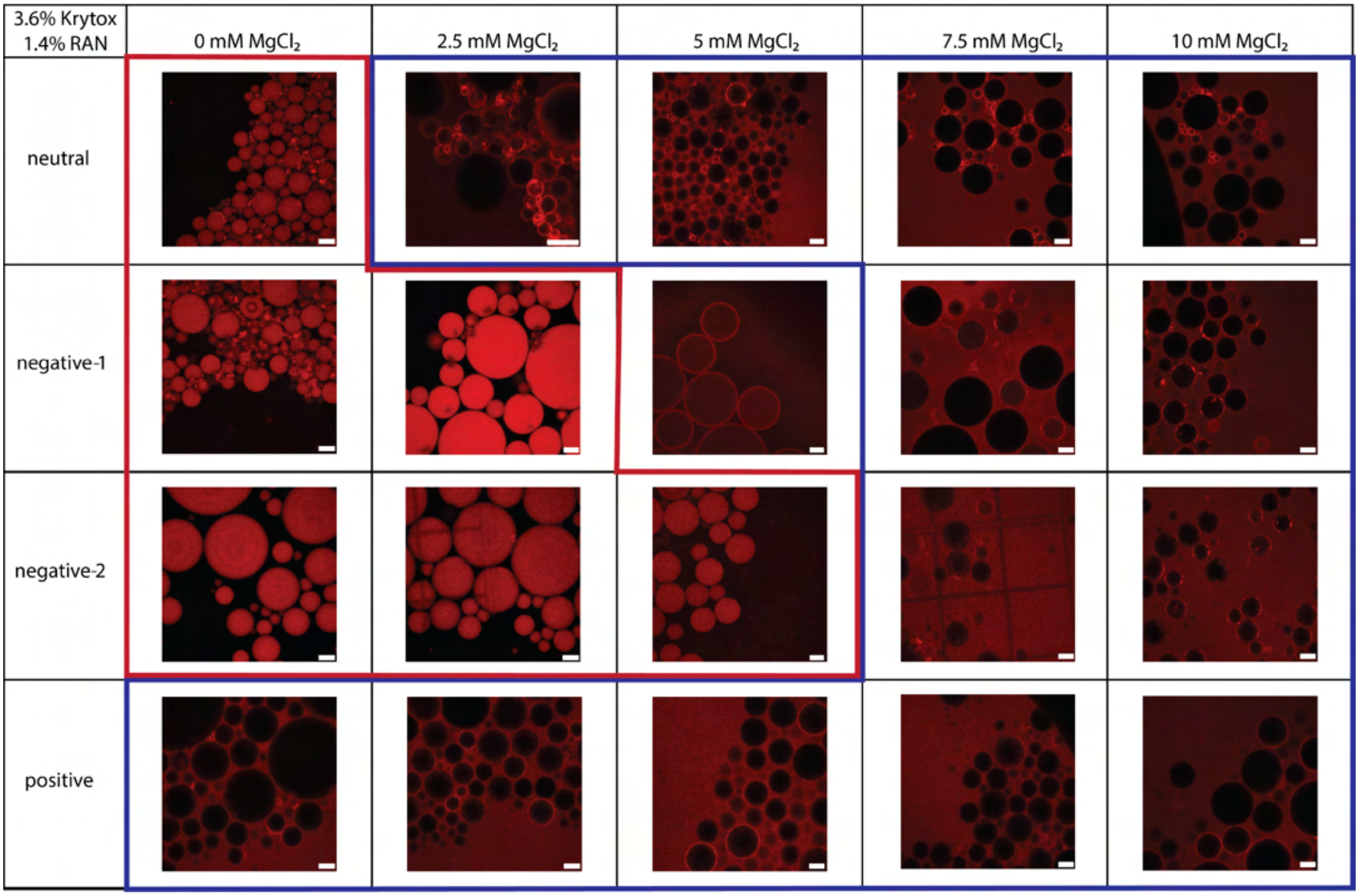

Figure S11: Droplets stabilized with 1.4% RAN and 5% Krytox surfactant. Red square indicates droplets where lipids do not assemble at interface. Blue square indicates samples were the fluorescence outside the droplet is higher than the fluorescence inside the droplet indicating serious leakiness (scale bar 50 µm).

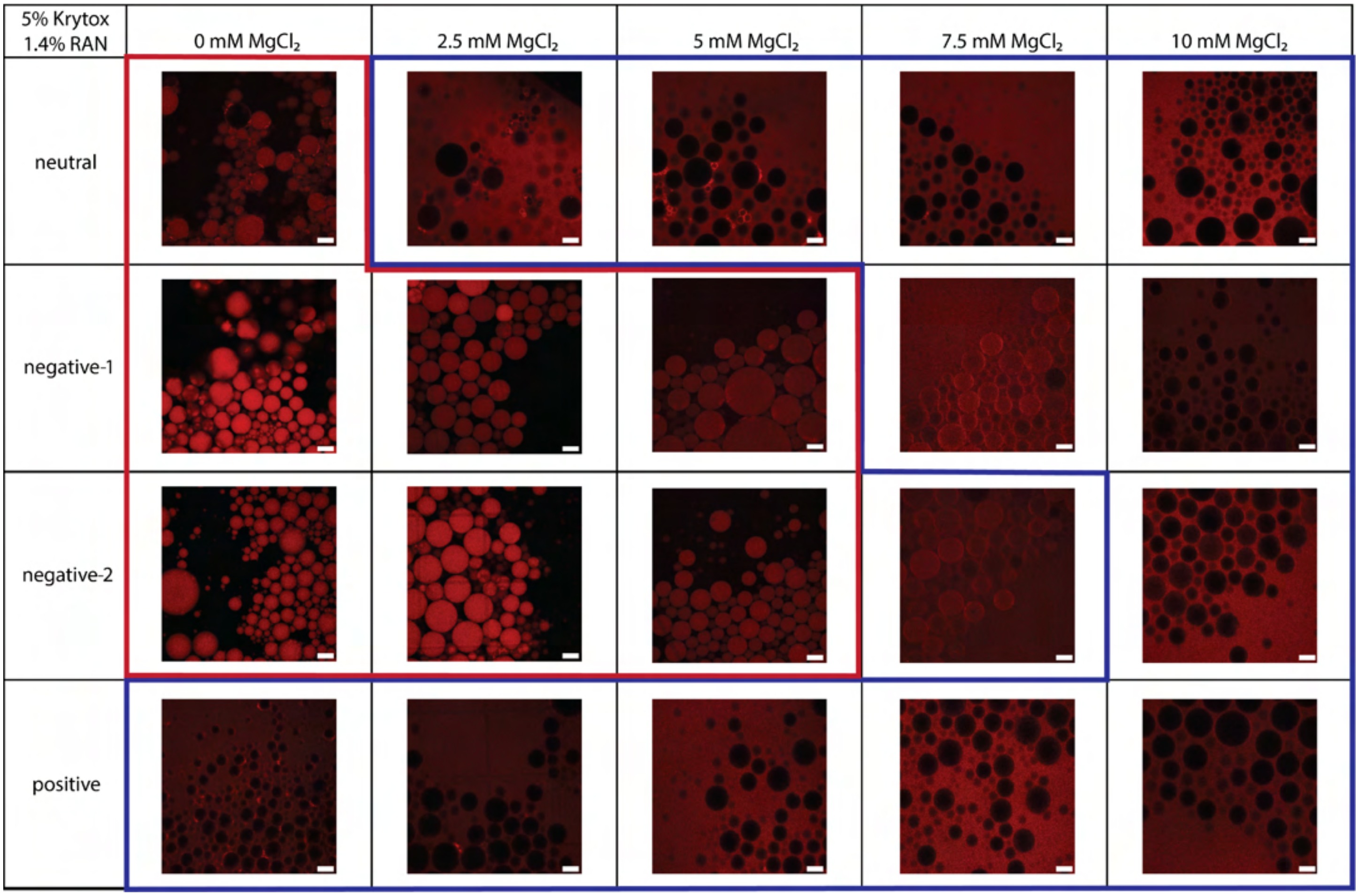

Figure S12: Droplets stabilized with 1.4% RAN and 3.6% Krytox surfactant. Red square indicates droplets where lipids do not assemble at interface. Blue square indicate sample where the fluorescence outside the droplet is higher than the fluorescence inside the droplet indicating serious leakiness (scale bar 50 µm).
